# Enhancing interlayer exciton dynamics by coupling with monolithic cavities via the field-induced Stark effect

**DOI:** 10.1038/s41565-025-01969-2

**Published:** 2025-07-17

**Authors:** Edoardo Lopriore, Fedele Tagarelli, Jamie M. Fitzgerald, Juan Francisco Gonzalez Marin, Kenji Watanabe, Takashi Taniguchi, Ermin Malic, Andras Kis

**Affiliations:** 1 Institute of Electrical and Microengineering, École Polytechnique Fédérale de Lausanne (EPFL), Lausanne, Switzerland; 2 Institute of Materials Science and Engineering, École Polytechnique Fédérale de Lausanne (EPFL), Lausanne, Switzerland; 3 Department of Physics, Philipps-Universität Marburg, Marburg, Germany; 4 Research Center for Electronic and Optical Materials, National Institute for Materials Science, Tsukuba, Japan; 5 Research Center for Materials Nanoarchitectonics, National Institute for Materials Science, Tsukuba, Japan

**Keywords:** Two-dimensional materials, Optical materials and structures

## Abstract

Optical microcavities provide a powerful and versatile framework for manipulating the dynamics of photonic emission from optically active materials through light recirculation. Spatially indirect interlayer excitons (IXs) exhibit broad tunability of their emission energy via the quantum-confined Stark effect. However, the electrical tunability of IXs has not been exploited in cavity-coupled systems until now. Here we modulate the detuning between the cavity resonance and the IX emission in a monolithic Fabry–Perot cavity using an applied vertical electric field. We reveal a simultaneous enhancement of both the emission intensity and lifetime of weakly coupled IXs when in resonance with the optical cavity owing to strong Purcell inhibition and cavity transparency effects. We further investigate the tunable momentum dispersion of coupled IXs through back-focal-plane imaging and explain our results by the cavity coupling of IX transition dipoles as supported by theoretical modelling. Our work demonstrates an integration effort enabling the versatile tuning of highly interacting IXs within monolithic cavities, revealing the attractiveness of electrically tunable IX cavity coupling for both fundamental studies towards exciton condensate manipulation and future integration of excitonic devices.

## Abstract (web summary)

This work advances excitonic optoelectronics by demonstrating electrically tunable cavity coupling of interlayer excitons in a van der Waals heterobilayer. Through electrostatic control, a 5-fold increase in exciton lifetime and a 50-fold boost in photoluminescence intensity have been achieved, which highlights the role of in-plane optical dipoles in weak coupling and provides momentum-resolved insights into exciton emission.

## Main

The exceptional optical properties of transition metal dichalcogenides (TMDCs)^1^ allow weak and strong light–matter coupling regimes^2^ to be accessed in cryogenic environments as well as at room temperature, enabling a prolific line of research on the modulation of exciton dynamics and the manipulation of exciton-polaritons^3–5^.

Type-II van der Waals heterobilayers, composed of different monolayer semiconductors, host spatially indirect Coulomb-bound electron–hole pairs called interlayer excitons (IXs), characterized by a permanent static electric dipole in the out-of-plane direction^6,7^. These IXs have attracted a growing interest owing to their broad emission energy tunability via the quantum-confined Stark effect^8,9^, exhibiting long lifetimes and diffusion lengths thanks to their dipolar nature^10–14^. Further research has focused on dipolar layer-hybridized species in TMDC natural homobilayers, which are also electrically tunable. They can be classified into two main kinds: (1) momentum-direct transitions with high oscillator strength^15–17^, which do not constitute the lowest energetic transition and emit weakly, and (2) momentum-indirect species that emit via their phonon replicas^11,18^.

Long-lived, strongly interacting dipolar ensembles provide an ideal platform for realizing Bose–Einstein condensates^19^. In particular, IXs in TMDC heterostructures with large binding energies were indicated as candidates for the realization of high-temperature Bose–Einstein exciton condensates^20^. Indeed, their properties have held promise towards the emergent spontaneous coherence of macroscopic order of IX ensembles^19,21^. In this context, the electrical control and enhancement of both emission and lifetime of IXs in TMDCs would be beneficial towards the realization of bright high-temperature Bose–Einstein condensates of long-lived dipolar excitons.

Until now, the research on IX coupling has mainly focused on applications such as lasing^22,23^ and investigations of commensurate heterobilayers grown by chemical vapour deposition in open cavity systems^24^. At the same time, the broad electrical tunability of IXs has not been explored in integrated optical microcavities. In fact, photonic crystals do not allow for dynamical detuning during device operation, and open cavities are not monolithic systems.

The spontaneous emission rate of excitons can be modified in the weak coupling regime through the Purcell effect^25^, trading off photoluminescence (PL) emission intensity with a longer lifetime^26^. Early observations in semiconductor quantum dots have reported the Purcell inhibition of the exciton spontaneous emission rate, that is, the increase of exciton lifetimes^27,28^. Thus, the IX lifetime would also be increased by Purcell inhibition, which typically comes at the cost of PL emission intensity. However, in the case of dipolar exciton ensembles, it is desirable to engineer a coupling condition where both the collected PL intensity is enhanced, and the lifetime of the interacting species is extended. The PL decrease induced by the Purcell inhibition can be overcome by exploiting the wavelength-dependent transparency of an optical cavity. While the combined enhancement of PL emission and lifetime has been shown for cavity-coupled indirect excitons in quantum well structures^29–32^, this was limited to a lifetime modulation of only 10% and up to ~2.5 ns (ref. ^30^).

In this work, we aim at reaching the combined strong enhancements of PL intensity and lifetime of IXs in a van der Waals platform by their optical cavity coupling. In particular, we achieve electrostatic control over the coupling of dipolar excitons by applying a vertical electric field in a van der Waals heterostructure, composed of a dual-gated WSe_2_/MoSe_2_ bilayer hosting IXs and embedded in optical microcavities composed of differential Bragg reflector (DBR) mirrors. By electrostatic modulation of the detuning between the IX emission energy and resonant cavity photons, we achieve the simultaneous enhancement of IX lifetimes (5×) and of their PL intensity emission (50×) when in resonance. Supported by theoretical modelling, we explain our results in terms of different coupling conditions for in-plane and out-of-plane optical transition dipoles, known to be present in the MoSe_2_/WSe_2_ moiré system^18,33^.

## Results

### Electrically tunable cavity-coupled IXs

Several TMDC combinations provide type-II alignments with IXs^3,6^. In our work, we chose MoSe_2_/WSe_2_ as an exemplary platform owing to the strength and the low inhomogeneous broadening of its interlayer PL emission at cryogenic temperatures^6^, together with the ultra-long lifetimes of the hosted IXs^3^. This choice was further motivated by the extensive literature on this platform, specifically concerning its angular emission pattern^34^ and optical transition dipoles^18,33^. We note that layer-hybridized species in WSe_2_ bilayers are also known to possess efficient PL emission and tunable character^11^, although their momentum-indirect nature could prove challenging for the study of tunable cavity coupling, which is out of the scope of this work. Other homobilayer TMDCs are characterized by momentum-direct hybrid species with high oscillator strengths^15,16^, but these do not constitute the lowest-energy transition, resulting in relatively weak PL emission^17^. Furthermore, owing to their intrinsic nature, the lifetimes of all these species are orders of magnitude lower (<1 ns) than type-II IXs.

**Fig. 1:**
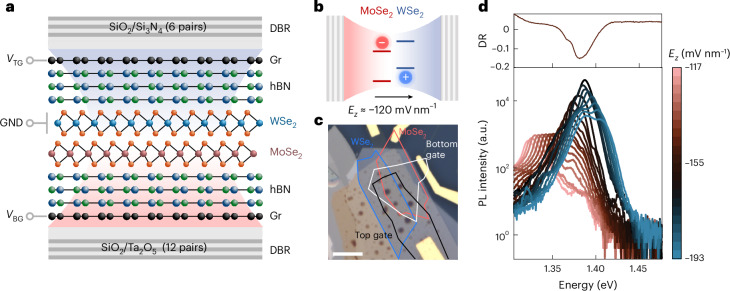
Electrically tunable cavity-coupled IXs. **a**, Schematic of the device structure, comprising a MoSe_2_/WSe_2_ heterobilayer encapsulated with hBN, with bottom and top graphene (Gr) layers. Bottom (*V*_BG_) and top (*V*_TG_) gate voltages are applied to the graphene layers, respectively, while the TMDC flakes are grounded (GND). The cavity consists of the bottom and top SiO_2_ layers and the van der Waals heterostack. The bottom DBR mirror comprises 12 pairs of Ta_2_O_5_–SiO_2_ layers, providing a wide reflectance window in the 800–1,000 nm range (Supplementary Fig. 2). The top DBR mirror consists of six pairs of SiO_2_–Si_3_N_4_ layers, each precisely matched at the optical path to correspond to half of the selected wavelength (*λ*/2). **b**, Illustration of type-II IXs in MoSe_2_/WSe_2_ in the condition of weak coupling with an applied electric field. **c**, Optical micrograph of device A, with highlighted flakes of WSe_2_ (blue), MoSe_2_ (red), bottom graphene (white) and top graphene (black). Scale bar, 10 µm. **d**, PL spectra (bottom) obtained from IXs at different electric fields in the full-cavity device A, excited by a laser power of 50 nW. In the vicinity of *E*_*z*_ ≃ −120 mV nm^−1^, the exciton emission is aligned with the differential reflectance (DR) dip (top). A notable enhancement of the collected PL intensity is present in the condition of exciton-cavity matching. This dataset is a subset of the full field-dependent spectra shown with a linear scale in Fig. 2a.

To exploit the quantum-confined Stark effect of IXs, we have fabricated dual-gated MoSe_2_/WSe_2_ bilayer structures fully encapsulated by hexagonal boron nitride (hBN), which we embedded in planar *λ*/2 cavities based on DBR mirrors^35^ (Methods and Fig. 1a). We used the optical transfer-matrix method to design the full stack and predict the energy mode and the quality factor (*Q*) of our structure. In Supplementary Note 4, we present more information on the chosen configuration of the DBR mirrors based on the losses in our system. The optical micrograph of the main structure used in this work (device A) is reported in Fig. 1c. We fabricated another structure (device B) to confirm our findings, and its optical micrographs are reported in Supplementary Note 1.

Before growing the top DBR pairs, we characterized the field-tunable emission of IXs and their dynamics in our platform (Supplementary Note 2). The lifetime of IXs on bottom DBR substrates is monotonically tuned with respect to the electric field *E*_*z*_ owing to the modulation of the electron–hole wavefunction overlap^14^. Then, we have designed our cavity so that its resonant energy falls within the same range as the tunable IX emission energy. After the top DBR deposition, we achieved an average quality factor of *Q* ~70 with variance ~30 within the whole double-gated heterostack and a cavity peak mode centred around 1.389 eV (Supplementary Note 3). In Fig. 1d, we show the field-tunable PL spectra obtained by modulating the detuning between the exciton (*E*_IX_) and cavity (*E*_C_) modes. In particular, from Fig. 1d, we extract an enhancement of the maximum IX peak intensity of a factor ~50 for cavity-coupled IXs (*E*_IX_ ≈ *E*_C_) with respect to uncoupled ones (*E*_IX_ < *E*_C_).

### Tunable inhibition of IX spontaneous emission rate

**Fig. 2:**
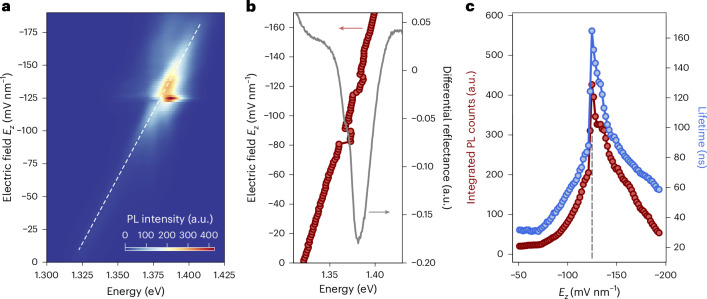
Tunable enhancement of emission and lifetime from cavity-coupled IXs. **a**, IX PL spectra as a function of the applied vertical electric field *E*_*z*_, obtained by exciting the structure with a 50 nW laser power. The dashed line corresponds to the linear quantum-confined Stark effect of IXs. A region of higher intensity is observed at the vicinity of 1.38 eV, corresponding to the cavity mode energy (Fig. 1d). We further show the field-dependent spectra in a waterfall plot in Supplementary Note 2, comparing it with the half-cavity case to better highlight the behaviour of the IX peak when entering the cavity transparency dip. **b**, Field-tunable position of the highest-emitting exciton peak energy (red). A shift of the brightest peak is found around –80 mV nm^−1^ owing to entrance of the IX tail into the transparency window. The shift around –120 mV nm^−1^ represents the first exciton-cavity resonance condition. The cavity mode is highlighted by the differential reflectance dip (grey). **c**, Total integrated IX PL intensity (red) and lifetime (blue) as a function of *E*_*z*_. Both intensity and lifetime exhibit a gradual increase for *E*_*z*_ < −50 mV nm^−1^, with a sharp peak at *E*_*z*_ ≃ −120 mV nm^−1^ followed by a gradual decrease. At resonance, as indicated by the dashed grey line, a 50-fold enhancement is observed for the integrated IX intensity, together with a 5-fold increase in lifetime.

Figure 2a shows the quantum-confined Stark effect of the IXs in our platform, excited by a pulsed picosecond diode laser (1.93 eV) with an average power of 50 nW and a repetition rate of 1 MHz. By fitting the Stark shift of the main IX peak, we estimate a dipole length of 0.5 nm, in agreement with previous reports^14,36^. We observe a sizable enhancement of the recorded PL as the exciton-cavity detuning is decreased. Furthermore, an apparent energy jump can be observed with the detuning at its minimum in Fig. 2b. For increasing electric field magnitudes with respect to the nominal resonance (*E*_*z*_ < −130 mV nm^−1^), the maximum energy peak position reverts back to the trend dictated by the linear Stark effect. This is attributed to the field-dependent tuning of our IX emission from lower to higher energies, causing the maximum PL peak to appear as shifted when the cavity transparency dip is reached (Supplementary Note 6).

We will hereafter refer to the structure without the top SiO_2_ and top DBR mirrors as the ‘half cavity’, and the complete device (Fig. 1a) as the ‘full cavity’ structures. Figure 2c shows the field-dependent integrated PL intensity and lifetime of the interlayer IX peaks in the full-cavity structure. As detailed in Supplementary Note 6, in the half-cavity structure, we observe a smooth decreasing PL intensity trend with respect to the applied electric field. However, in the full-cavity structure, we measured an enhancement of intensity for electrostatic fields that minimize exciton-cavity detuning. In particular, an increase in PL emission is observed for fields *E*_*z*_ ≤ −50 mV nm^−1^, with a peak around *E*_*z*_ ≃ −120 mV nm^−1^ followed by a gradually decreasing tail. To understand the asymmetric trend of the integrated PL intensity recorded from our system, we used optical transfer-matrix method simulations of the far-field emission of ideal dipoles in the energy range of our IXs (Methods). As further discussed in the following sections and in Supplementary Note 9, we ascribe such asymmetry to the angle-dependent emission of cavity-coupled in-plane IX optical transition dipoles.

Furthermore, we measured the field-dependent dynamics of our interlayer ensembles by time-resolved PL, as shown in Fig. 2c. Before the top cavity growth, we recorded lifetimes in the range of tens of nanoseconds. In particular, the trend of decreasing lifetime with increasing *E*_*z*_ in the half-cavity structure (Supplementary Fig. 2) is directly related to the modulation of the electron–hole wavefunction overlap by the quantum-confined Stark effect, as in previous reports^11,14,37^. In the full-cavity structure, we observe a sharp increase of the IX lifetime under the exciton-cavity matching condition, with a fourfold increase with respect to the half-cavity excitons (Supplementary Fig. 8d). Therefore, our structure exhibits a combined enhancement of both the extracted PL intensity and the lifetime of IX ensembles when tuned to the cavity resonance.

We explain the obtained lifetime enhancement by a Purcell inhibition of the IX spontaneous emission rate (that is, increase in IX lifetime). The main factors that can contribute to a sizable Purcell inhibition are spatial misalignment^38^, spectral detuning^39^ and the depletion of photonic states owing to the optical microcavity^40^. Since we use a planar *λ*/2 cavity, the spatial detuning can be assumed to be negligible, while the quantum-confined Stark effect allows us to minimize the spectral detuning. By comparing with the half-cavity structure (Supplementary Fig. 8d), we confirm that the observed lifetime enhancement is a result of the modulation of the radiated photonic mode density, thus allowing a nontrivial simultaneous enhancement of the collected PL intensity. Therefore, we attribute the observed lifetime trend to the discontinuity of photonic mode density at the resonant condition for weakly coupled in-plane IX transition dipoles^41,42^ (Supplementary Note 9).

### Field-effect tuning of momentum-resolved IX emission

**Fig. 3:**
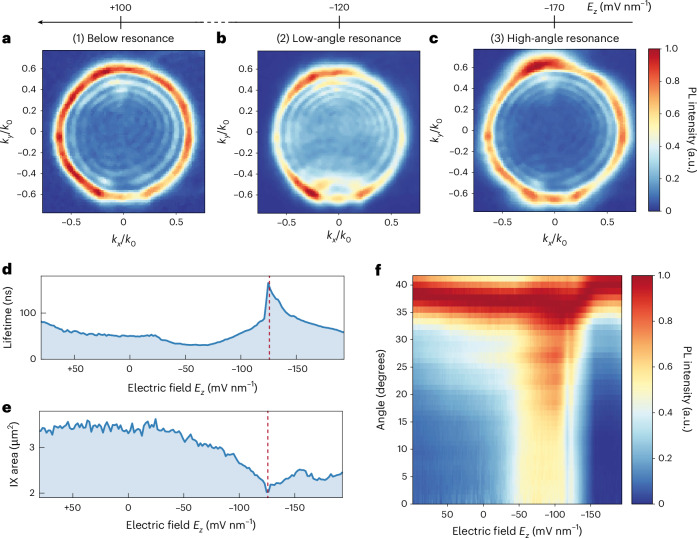
Dispersion of cavity-coupled IXs in momentum space. **a**–**c**, Momentum-resolved IX emission obtained by BFP PL spectroscopy at electric field strengths of *E*_*z*_ ≃ 100 mV nm^−1^ (**a**), −120 mV nm^−1^ (**b**) and −170 mV nm^−1^ (**c**), respectively. All measurements were obtained under a 50 nW laser excitation. High-momentum components dominate the signal for **a** and **c**, while low-momentum PL emission arises in the case of **b**. The complete field-dependent BFP dataset is shown in Supplementary Video 1. BFP images are normalized at each electric field to show the different components in momentum space at all exciton-cavity detuning conditions. **d**,**e**, Field-dependent lifetime (**d**) and effective IX diffusion area (**e**). The data in **d** are an extension of that shown in Fig. 2c. In agreement with previous reports^11,13^, we define the IX effective diffusion area as the region of the IX emission cloud in space with PL intensity above 1/*e* of its maximum. The IX emission cloud is recorded by a CCD camera, as described in Methods. The dashed red lines indicate the electric field value of lowest IX effective diffusion area, as well as highest PL intensity and lifetime. **f**, Field-dependent angular emission is obtained by radially averaging the measured BFP images at each electric field. The recorded PL emission is normalized at each electric field. The enhancement of the low-angle emission components is observed for electric fields in the range −130 mV nm^−1^ < *E*_*z*_ < −50 mV nm^−1^, corresponding to region (2).

To investigate the radiation pattern of cavity-coupled IXs in our monolithic DBR system in momentum space, we performed Fourier imaging of the back-focal-plane (BFP) PL emission with respect to the applied electric field. Figure 3a–c shows the momentum-resolved emission of IXs at three different fields. *k*_*x*_ and *k*_*y*_ represent the *x* and *y* components of the in-plane photon wavevector *k*_0_ sin *θ*, where *θ* is the emission angle and *k*_0_ is the photon wavevector in air.

We define three main regions of the applied electric field: (1) below resonance (*E*_*z*_ > −50 mV nm^−1^), (2) low-angle resonance (−50 > *E*_*z*_ > −130 mV nm^−1^) and (3) high-angle resonance (*E*_*z*_ ≤ −130 mV nm^−1^). We observe a dominant high-angle emission in the momentum dispersion below cavity resonance (*E*_*z*_ ≈ 100 mV nm^−1^; Fig. 3a). We note that a non-negligible signal around zero momentum is also present. Moreover, we observe a sizable enhancement of low-angle emission when in the exciton-cavity mode matching condition (*E*_*z*_ ≈ −120 mV nm^−1^; Fig. 3b), concurrently with the recorded enhancement of both PL intensity and lifetime (Fig. 3d). When further increasing the applied electric field magnitude, the emission pattern reverts back to a situation of dominant high-angle emission, as shown in Fig. 3c (*E*_*z*_ ≈ −170 mV nm^−1^).

To further highlight the field dependence of the momentum-resolved emission in our system, we calculate a radial average of the *k* components in the BFP images with respect to *E*_*z*_ (Fig. 3f and Supplementary Video 1). These results reveal a change in the angular emission of cavity-coupled IXs in correspondence with the enhancement of their emitted PL intensity and lifetime. Moreover, as the electric field decreases from −50 mV nm^−1^ to −130 mV nm^−1^, time-integrated imaging of the spatial distribution of IX emission shows a notable reduction in the IX diffusion area and simultaneous increase of IX lifetime, as illustrated in Fig. 3d–f. This coincides with a radiative enhancement of low-momentum IXs, suggesting a potential mechanism for photonic IX localization.

WSe_2_/MoSe_2_ heterobilayers are known to host a rich excitonic platform owing to the moiré potential arising from the atomic registry between the two layers^18,33,43,44^. In particular, the moiré superlattice in incommensurate WSe_2_/MoSe_2_ heterobilayers is expected to give rise to both in-plane and out-of-plane IX transition dipoles of comparable strength^18^, where only the in-plane transition dipoles are expected to couple optimally with planar cavities^39^. Thus, different IX species with different group velocities and emission profiles are expected to coexist. The exciton group velocity *v*_g_ can be defined based on the Wannier function approach for both monolayer TMDCs^45^ as well as for IXs in moiré heterobilayers^46^. Thus, we explain our results by the enhancement of the emission of low-*v*_g_ IXs when in resonance, inducing an effective IX cloud area reduction with respect to off-resonance or high-angle resonance conditions. In fact, at resonance, the IX species with lower group velocity must preferentially couple into low-angle (low-momentum) optical modes^45^. When the electric field is between −130 mV nm^−1^ and −160 mV nm^−1^, the visualized IX effective diffusion area expands, corresponding with an increase in emission from higher-momentum IXs. Finally, for *E*_*z*_ ≤ −160 mV nm^−1^, the IX emission momentum remains dominated by high-momentum components, while a pronounced decrease in IX lifetime correlates with the observed decrease in the IX diffusion area.

### IX transition dipoles and cavity coupling

**Fig. 4:**
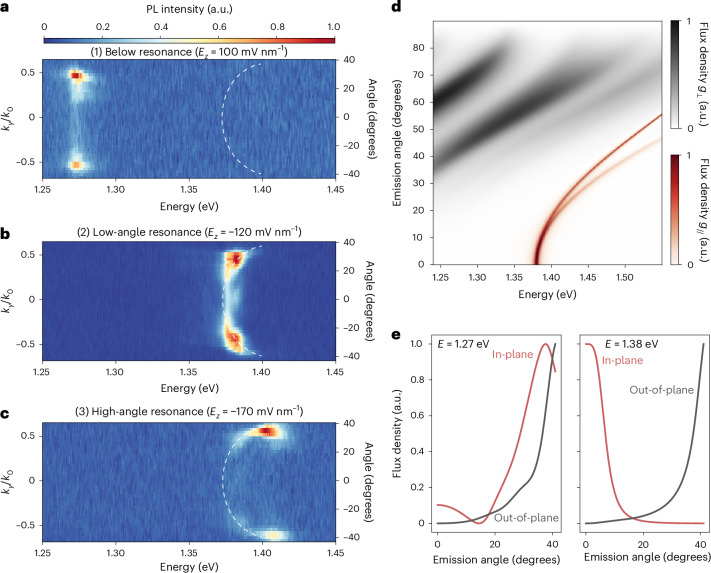
Field-dependent angular emission of IX transition dipoles in an optical microcavity. **a**–**c**, Energy-resolved IX emission obtained by the *k*_*y*_ component of BFP PL spectroscopy at electric fields *E*_*z*_ ≃ 100 mV nm^−1^ (**a**), −120 mV nm^−1^ (**b**) and −170 mV nm^−1^ (**c**), respectively. All measurements were obtained under 50 nW laser excitation. In **a**, high-angle emissions dominate the signal, with a non-negligible component at low angles. When approaching the resonant condition (**b**), low-angle emissions are enhanced, with residual components still present at higher angles. Moving towards higher electric fields (**c**), a progressive quasi-parabolic shift of the emitted PL is observed towards higher angles (Supplementary Video 2). The dashed white lines are guides to the eye following the field-dependent angular dispersion of the IX PL. **d**, Transfer-matrix simulations of the angular emission pattern of ideal in-plane (*g*_//_) and out-of-plane (*g*_⊥_) dipoles within our structure with respect to their emission energy. The relative intensity of the emission flux density is normalized for both dipolar species. No coupling condition is achieved for out-of-plane dipoles. Instead, a sharp rise in the in-plane dipole emission is present at the nominal cavity mode (1.38 eV). With increasing energies, the cavity coupling condition is still obtained for higher angles of emission of the in-plane dipole, thus yielding a parabolic trend at low angles, becoming linear at higher energies. **e**, Left: the emission in the below-resonance range (*E* ≃ 1.26 eV), retrieved from a linecut in **d**, shows a non-negligible flux density at low angles (*θ* < 20°) for the in-plane dipole, followed by a dominant signal at higher angles (*θ* > 40°). The emission from the out-of-plane dipole gives a sizable flux density at high angles. This is in agreement with the observed emission in **a**. Right: the emission at exciton-cavity resonance (*E* ≃ 1.38 eV) is strong in the vicinity of 0° for the in-plane dipole owing to efficient cavity coupling. By contrast, a high-angle signal is still present for the out-of-plane dipole. Thus, the sudden change to 0° emission in the exciton-cavity matching condition of **b** is related to the selective cavity coupling of in-plane IX transition dipoles. The trend for *E* > 1.38 eV in **d** is consistent with the resonance observed in **c**.

To understand the tunability of our cavity-coupled IX emission, in Fig. 4a–c, we show the energy-resolved PL of IXs in our platform with respect to their angular pattern (Methods), obtained from the *k*_*y*_ component of the BFP data. In particular, Fig. 4a–c is representative of regions (1) to (3), respectively. The complete energy-resolved field-dependent dataset is shown in Supplementary Video 2, reporting the linear Stark shift of the IX emission in energy and the corresponding evolution of its angular pattern. In region (1), we observe dominant emission at high angles for all IX energies, as previously shown by the corresponding BFP measurements (Fig. 3a). However, for conditions (2) and (3) in Fig. 4b,c, respectively, the emitted intensity follows a quasi-parabolic trend towards higher angles.

A notable enhancement in the low-angle emission in region (2), together with the quasi-parabolic trend in (2) and (3), is also reported in Supplementary Fig. 9 for another position of the heterostructure in device A, as well as in Supplementary Fig. 11 for device B. We note that no substantial difference is observed at different excitation powers, as shown by the characterization in Supplementary Fig. 9 conducted at 0.9 mW. These results show that the achieved field tunability of the IX angular emission pattern is independent of intrinsic heterostructure region-to-region variations.

To understand the observed energy dispersion of coupled IX emission, we need to consider the presence of both in-plane and out-of-plane transition dipoles in our heterobilayer^18^, as mentioned in the previous section. In particular, although the IX energy of emission can vary based on the moiré periodicity, an emitting interlayer transition in a given TMDC heterobilayer with little atomic mismatch will feature a non-negligible coupling with in-plane and out-of-plane photon modes, with properties that vary across the moiré cell based on the atomic registry. In the case of WSe_2_/MoSe_2_, both spin-singlet and spin-triplet transitions are expected to possess both in-plane and out-of-plane transition dipoles within a moiré cell^18^. Therefore, we have performed optical transfer-matrix simulations of ideal emitters in our structure comprising both out-of-plane and in-plane optical dipoles as a function of the optical dipole emission energy. In Fig. 4d, we show the emitted intensity flux obtained for a wide range of emission energies, mimicking the energy span covered by our field-tunable interlayer species. At energies lower than the cavity mode (*E* < 1.38 eV), both in-plane and out-of-plane dipoles give emitted fluxes with maximal intensities at high angles (>30°), as further highlighted in Fig. 4e. The presence of a sizable lobe of the flux density at low angles for the in-plane transition dipole is aligned with the non-negligible signal that we experimentally observe at low momentum off resonance (Fig. 4a). Thus, we attribute the observed emission in the region (1) of the field-tunable BFP measurements to the superposition of both in-plane and out-of-plane IX transition dipoles of different local atomic registries^34^. By contrast, we observe a sharp increase of the in-plane dipole emission when reaching the cavity mode, as in region (2). Figure 4d shows that, for increasing energies, cavity coupling is achieved for in-plane dipoles at higher angles, exhibiting a parabolic trend in the energy dependence of coupled emission, confirming our observations in region (3). We note that our collection is limited to a partial range of angles (±40°; Methods), which is nonetheless wide enough to be covered by our simulations. In Supplementary Note 8, we mimic the exciton emission energy tunability by convoluting the simulated emission of an in-plane dipole in our structure with Lorentzian broadening. As a result, Supplementary Fig. 14 gives a theoretical representation of the tunable cavity coupling of in-plane IX transition dipoles in our structure.

We note that the large structural changes owing to mesoscopic reconstruction have been shown to strongly impact the spectroscopic signatures of IXs^47^. While we acknowledge the possibility that bubbles and morphological inhomogeneities could induce mesoscopic reconstructions in our samples, any arbitrary IX transition dipole can always be described as a superposition of two orthogonal transition dipoles^48^ (Supplementary Note 9), thus further motivating our theoretical treatment (Fig. 4d). This is corroborated by the qualitatively consistent behaviour of cavity-coupled IXs between different positions within the same device and across different devices (Supplementary Notes 6 and 7). Additional information on the spatial analysis of the IX emissions in device A is provided in Supplementary Note 6.

On the basis of the previous discussion, we explain the sharp increase in emission intensity at exciton-cavity resonance based on the change in the angular emission pattern of weakly coupled in-plane IXs owing to cavity transparency. By contrast, at all electric field strengths, the out-of-plane IX dipoles do not efficiently couple to the cavity modes supported by our structure.

## Conclusion

We have obtained the field-tunable simultaneous enhancement of the emitted IX intensity and lifetime when the IX emission is tuned to the optimal cavity coupling, a highly desirable outcome towards efficient excitonic devices with an electrically tunable coupled emission. Our results are corroborated by simulations of emitting dipoles in our structure, showing that in-plane IX transition dipoles are responsible for the observed weak coupling in our planar monolithic microcavity system. Our work shows the electrically tunable cavity coupling of IXs in a van der Waals heterobilayer within a monolithic system, representing a paradigm shift in the research on cavity-coupled emission of dipolar excitons.

We expect our work to motivate further investigation into other platforms exhibiting field-tunable interlayer species, such as momentum-direct hybrid excitons in TMDC homobilayers, ultimately leading to the electrical control of the strong cavity coupling of dipolariton ensembles^49^. Furthermore, the electrical switching of interacting IXs and their cavity coupling could bring the field one step closer to the realization of Bose–Einstein condensation of field-tunable propagating ensembles.

## Methods

### Device fabrication

The bottom DBR (Laseroptik) was fabricated by successive deposition of Ta_2_O_5_ and SiO_2_ layers, followed by a SiO_2_ layer as the bottom part of the central cavity structure. Graphene has been used for both top and bottom gates to minimize optical losses. For all devices, the bottom graphene gate was obtained by direct exfoliation of graphite (NGS) on such substrates. The top and bottom hBN layers were each chosen to be 30 nm thick to align the cavity electric field maximum with the centre of the heterostructure. All flakes of device A, except for the bottom graphene, were transferred by a two-polymer wet transfer method, where the bottom polymer was dissolved in a solvent and the top polymer was left free-floating to be transferred. After wet transfer, the top polymer layer was cleaned in acetone. The bottom hBN, monolayer WSe_2_ (HQ Graphene), monolayer MoSe_2_ (HQ Graphene), top hBN and top graphene layers were successively transferred using the wet method on top of the bottom exfoliated graphene. Instead, the heterostructure of device B was fabricated with a dry-transfer technique using a polycarbonate membrane over a PDMS substrate. In this case, WSe_2_, MoSe_2_ and hBN were exfoliated on PDMS (gelpak) and SiO_2_ substrates. The top hBN layer was picked up from SiO_2_ with the polycarbonate membrane and was then used to pick up monolayer WSe_2_ and MoSe_2_ from gelpak and bottom hBN from SiO_2_. This four-layer heterostack was then released on top of the exfoliated bottom graphene by a progressive adhesion to the substrate while increasing the temperature above 150 °C. Also, in this case, the top graphene was deposited using a wet transfer technique. All electrical contacts to the graphene gates and to the TMDCs of all devices were fabricated using electron-beam lithography and metal evapouration (2 nm Ti/80 nm Au). All structures were annealed for 6 h at a temperature of 340 °C in high vacuum (10^−6^ mbar). The top structure of oxide layers was then deposited using plasma-enhanced chemical vapour deposition, with the thickness of the first SiO_2_ layer to match the *λ*/2 cavity condition, and the top SiO_2_/Si_3_N_4_ DBR layers to match the *λ*/4 wavelength value. The twist angles between the TMDC layers in devices A and B were estimated from second harmonic generation measurements, as described in Supplementary Note 11, revealing alignments of 1.7° ± 0.3° (R-type) and 1.3° ± 0.4° (H-type) for devices A and B, respectively.

### Optical measurements

All optical measurements were performed in a vacuum environment at 4.2 K, unless stated otherwise, in a He-flow cryostat. IXs were excited with a confocal microscope while the emitted photons were collected through the same objective with a working distance of 4.5 mm and a numerical aperture of NA = 0.65. Optical pumping was achieved with a continuous-wave 640 nm diode laser (PicoQuant, LDH-IB-640-M) focused to the diffraction limit (spot full width at half maximum of 1.2 μm) for steady-state measurements. For μPL spectral measurements, the emitted light was filtered by a 700 nm long-pass edge filter and then acquired using a spectrometer (Princeton Instruments SpectraPro 500) and recorded with a charge-coupled device (CCD) camera (Princeton Instruments, Blaze 400-HR/HRX). For differential reflectance measurements, a stabilized white light lamp (Thorlabs SLS202L) focused to a 2.0 μm FWHM spot has been used for excitation. The reflected signal from the sample was then recorded by the same spectrometer and camera used for μPL spectroscopy. Spatial imaging of the IX emission was captured by a CCD camera (Andor Ixon) with an 800 nm long-pass edge filter that removes both the laser line and the intralayer emission from WSe_2_ and MoSe_2_. For time-resolved measurements, the same solid-state laser is driven in pulsed mode, achieving pulse widths lower than 160 ps at a 1 MHz repetition rate. The collected photons are sent to an APD (Excelitas Technologies, SPCM-AQRH-16). The output of the APD is connected to a time-correlated photon-counting module with a resolution of 12 ps r.m.s. (PicoQuant, PicoHarp 300), which measures the arrival time of each photon. For the measurements in this work, we set the time bin to 16 ps. The single-photon timing resolution of the APD is ~350 ps, which is the main time limitation for this set-up. BFP imaging and spectroscopy were performed by inserting a plano-convex lens between the objective and the focusing lenses of the CCD camera (Andor Ixon Ultra) and spectrometer, respectively. The conversion from momentum to angular emission is done following previous works in the literature^50^. In particular, NA = *n* sin(*θ*_max_), where *n* is the refractive index of the medium (air in our case) and *θ*_max_ is the maximum emission angle recorded from our system. In radial coordinates, for a generic angle *θ*, we have $\sin \theta \propto r=\sqrt{{x}^{2}+{y}^{2}}$, based on a multiplication constant that we obtain from the known NA value.

### Theoretical modelling of dipolar emission

Theoretical modelling of the emission characteristics of a dipole source placed within the microcavity structure was performed using a combination of the transfer-matrix method (Ansys Lumerical STACK) and the finite-difference time-domain method^51^ (using the open-source software package MEEP^52^). Excellent agreement between the two methods was found (Supplementary Note 8). To model the emission of incoherent excitons with an in-plane dipole orientation, we separately simulate classical dipole sources aligned along the *x* and *y* directions, and then sum the resulting field intensities.

## Online content

Any methods, additional references, Nature Portfolio reporting summaries, source data, extended data, supplementary information, acknowledgements, peer review information; details of author contributions and competing interests; and statements of data and code availability are available at 10.1038/s41565-025-01969-2.

## Supplementary information

Supplementary Information Supplementary Notes 1–11, Figs. 1–20 and text references.

Supplementary Video 1 Field-dependent BFP measurements of IX emission in device A.

Supplementary Video 2 Field-dependent energy-resolved measurements of IX angular emission in device A.

## Data Availability

The data that support the findings of this study are available via Zenodo at 10.5281/zenodo.15355928 (ref. ^53^).
