# Supplementary material for: Enhancing interlayer exciton dynamics by coupling with monolithic cavities via the field-induced Stark effect
Source: Nat Nanotechnol. 2025 Jul 17;20(10):1412–8. doi: 10.1038/s41565-025-01969-2 (PMC12534180; doi:10.1038/s41565-025-01969-2)
Supplement: Supplementary file 1 — Supplementary Notes 1–11, Figs. 1–20 and text references. [file 41565_2025_1969_MOESM1_ESM.pdf]

nature nanotechnology

Supplementary information

https://doi.org/10.1038/s41565-025-01969-2

Enhancing interlayer exciton dynamics by coupling with monolithic cavities via the field-induced Stark effect

In the format provided by the authors and unedited

1. Device B structure 2
2. Dual-gated interlayer excitons for the half-cavity structure 2
3. Quality factor statistics 4
4. DBR cavity simulations 5
5. Field-dependent lifetime in device A 7
6. Further characterization of device A 7
7. Characterization of device B 11
8. Simulations of cavity-coupled dipolar emission 13
9. Transition dipole orientation and weakly-coupled IX lifetime 17
10. Experimental Setup 18
References 20

1. Device B layout

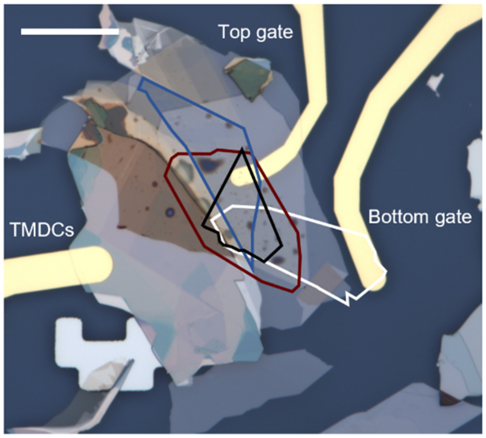

Supplementary Fig. 1. Optical micrograph of the dual-gated hBN-encapsulated device B before top DBR and top SiO_2 growth (half-cavity structure). The WSe_2 (blue), $MoSe_2$ (red), top graphene (black) and bottom graphene (white) layers are highlighted by contour lines. The metal contacts to the TMDCs layers and to the top and bottom gates are made of Ti/Au. Scale bar: 25 μm.

The layout of device B is reported in Supplementary Fig. 1, with all flakes of interest highlighted by contour lines. It was fabricated as described in the Methods section in the main text. The optical micrographs show large overlapping areas between all highlighted flakes. Both devices were subsequently closed with top DBR mirrors grown by PECVD. All hBN thicknesses were measured by AFM scans, with $t^{A}_{topBN} = 31\ \text{nm} = t^{A}_{topBN}$, and $t^{B}_{topBN} = 26\ \text{nm}$ and $t^{B}_{botBN} = 27\ \text{nm}$.

2. Dual-gated interlayer excitons for the half-cavity structure

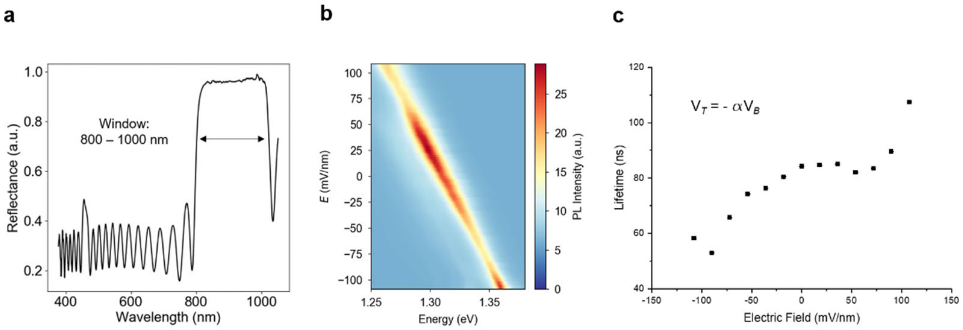

Supplementary Fig. 2. a, Reflectance spectrum of the bottom part of the cavity structure, comprising half cavity SiO_2 / bottom DBR / substrate SiO_2 / Si (p+), which is the stack of materials lying below the van der Waals heterostructure. The reflectance window (800−1000 nm) is highlighted. **b,** Quantum-confined Stark effect of IXs in the half-cavity device, comprising the van der Waals heterostructure and the bottom part of the cavity structure, with window measured in (a). **c,** IX lifetime as a function of the applied electric field in the half-cavity device. All lifetime measurements are taken with a low excitation power of 70 nW.

Supplementary Fig. 2b shows the quantum-confined Stark effect on interlayer excitons measured from the heterostructure of the half-cavity structure of device A. To induce a vertical electric field with no extrinsic electrostatic doping we apply top and bottom gate voltages as

$V_T = -\alpha V_B$, where $\alpha = t_{topBN}/t_{botBN}$ is the ratio between the top and bottom hBN thicknesses. Thus, the electric field is estimated as:

$$E_z = \frac{V_B - V_T}{d_{tot}} \cdot \frac{\varepsilon_{hBN}}{\varepsilon_{hb}} = \frac{V_{DIFF}}{d_{tot}} \cdot \frac{\varepsilon_{hBN}}{\varepsilon_{hb}} \quad (1)$$

where $\varepsilon_{hb} \approx 7.5$ is the dielectric permittivity of the $WSe_2/MoSe_2$ heterobilayer area, $\varepsilon_{hBN} \approx 4$ and $d_{tot} = d_{hBN}^{top} + d_{hBN}^{bot} + d_{WSe_2} + d_{MoSe_2}$, where $d_{WSe_2} + d_{MoSe_2} \simeq 1.3$ nm.

We extract the IX dipole length from the Stark shift in Supplementary Fig. 2b as follows:

$$d = -\frac{1}{e} \cdot \frac{\partial \Delta E_{hIX}(F_{el})}{\partial F_{el}} \quad (2)$$

where $\partial \Delta E_{hIX}(F_{el})/\partial F_{el}$ is the variation of the dominant IX peak energy as a function of the applied field. We obtain $d \sim 0.5$ nm, in line with previous reports on IXs in $WSe_2/MoSe_2$[1–3]. The same dipole length extraction is also performed in the cavity-coupled case (Fig. 2b), obtaining the same result as before the top DBR growth. Supplementary Fig. 2d shows the field-dependent lifetime of IXs in device A before growing the top DBR structure, with trends that are well aligned with previous reports[2].

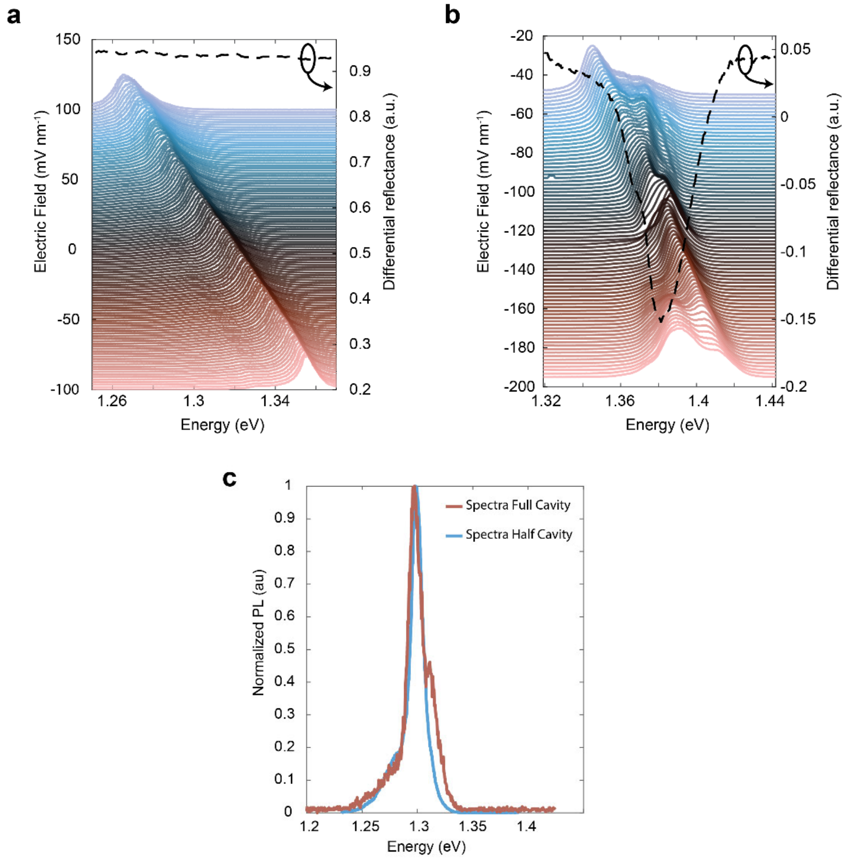

Supplementary Fig. 3. a-b, Waterfall plots of the field-dependent emission of IXs in the half-cavity (a) and full-cavity (b) structures (position A). **c,** PL spectra of the IX emission off-resonance in the full-cavity structure (red) compared with the equivalent energetic emission in the half-cavity case (blue).

In order to compare the field dependence of the IX emission in the half-cavity and full-cavity structures, Supplementary Fig. 3a and Supplementary Fig. 3b display waterfall plots of a subset of data from Supplementary Fig. 2b and Fig. 2a, respectively. In particular, Supplementary Fig. 3b highlights the enhancement of the IX PL tail as it approaches the reflectance dip, followed by the resonant enhancement at the cavity mode. Furthermore, Supplementary Fig. 3c shows the off-resonant IX emission obtained before and after the top DBR growth, confirming that the emission characteristics of our platform away from the cavity resonance remains unperturbed by the mirror deposition.

3. Quality factor statistics

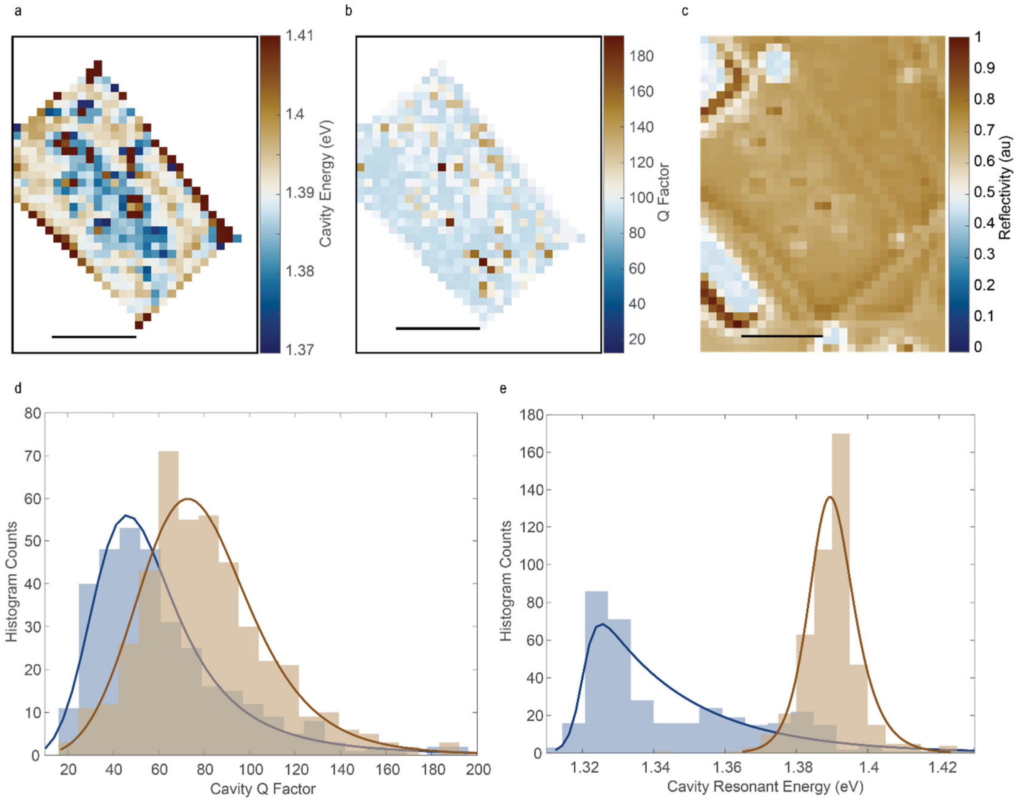

Supplementary Fig. 4. a-b, Peak energy (a) and quality factor (b) of the main cavity reflectance dip (Q_2) obtained along the heterostructure area by white-light reflectance measurements. **c**, In order to reconstruct the bare heterostructure area, we illuminated the same spots used for the white-light reflectance measurements with a low-power laser light. From the reflectance intensity, we extracted information on the spatial features of the heterostructure, allowing us to locate the different flakes and the full heterostructure area. **d-e,** Histogram plots of the Q factor and the energy position of the cavity reflectance dips throughout the area featuring both top and bottom hBN layers. Scale bars are 10 μm in every map.

The complexity of our heterostructures induces non-idealities in the growth of the top layers by PECVD. In particular, even though high conformity is achieved by PECVD growth[4], the presence of bulk areas and dielectric bubbles can induce shifts in the energy and the quality factor of the full-cavity structure. Supplementary Fig. 4 displays the statistics of the Q factor and the energy position of the reflectance dip obtained within the wavelength window of interest. We observe a reflectance dip that is best described by two Gaussian fits. Thus, in order

to draw a statistical analysis of our growth along the heterostructure, we define Q_1 and Q_2 as the Q-factors of the two main dips, with the respective peak energy positions E_1 and E_2. Their statistics are shown as histograms in Supplementary Figs. 4d-e. These statistics are obtained along the heterostructure area comprising top and bottom hBN, the main components dictating the resonance wavelength of the full-cavity structure. We extract average Q factors of $Q_1 = 45.4 \pm 23.4$ and $Q_2 = 71.8 \pm 28.5$ for peaks centered at $E_1 \simeq 1.326 \pm 0.013$ eV and $E_2 \simeq 1.390 \pm 0.007$ eV, respectively. For our study on IXs, we focus on heterostructure areas dominated by the Q_2 peak. All results shown in the main text, as well as those for a different point of Supplementary Note 6, refer to positions with reflectance dips located at 1.38 eV.

4. DBR cavity simulations

We performed transfer-matrix method (TMM) simulations to predict and understand the cavity mode properties of our structure[5]. In particular, we evaluated the use of graphene gates and their impact on absorption losses.

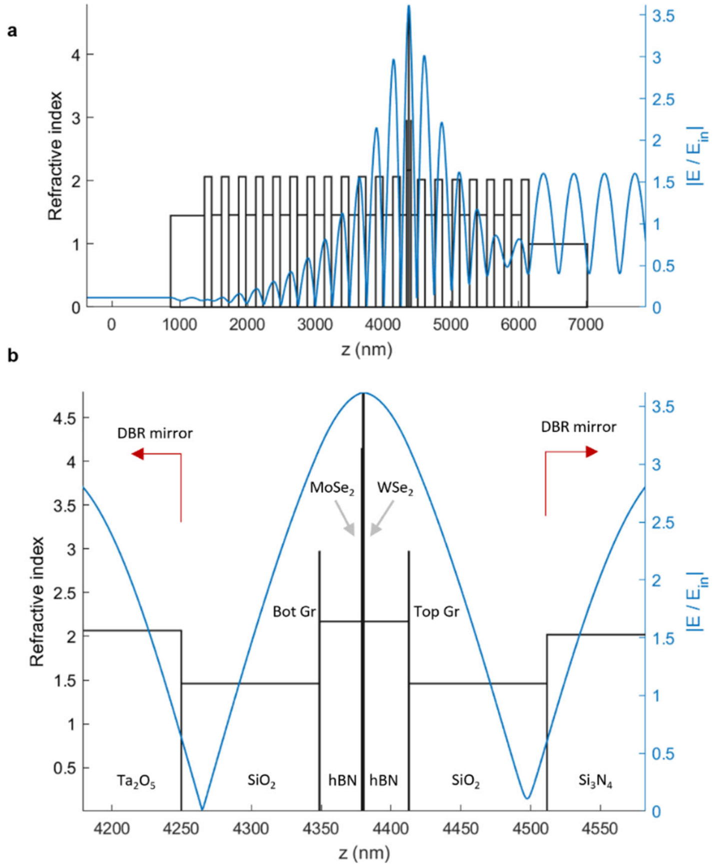

Supplementary Fig. 5. a, Electric field enhancement in the heterostructure area at a resonant wavelength of 900 nm with top and bottom hBN layers of 30 nm. **b**, Close-up from (a) on the refractive indexes of the materials in the cavity region. A slight deviation of the nodes in the $\lambda/2$ condition is obtained with respect to an ideal SiO_2 cavity due to the fact that both hBN and SiO_2 are contributing as cavity dielectrics.

In our simulations, we included 30 nm of hBN for both top and bottom layers, consistent with our experimental conditions, and included all refractive indexes of interest[6–8]. The cavity was designed to have an optical thickness of $\lambda/2$ with the thickness of the SiO_2 layers in the cavity

region adjusted accordingly. The number of bottom DBR pairs (Ta_2O_5/SiO_2) was set to 12. In Supplementary Fig. 5, we show the electric field distribution obtained for 6 top DBR pairs (SiO_2/Si_3N_4) at the resonant wavelength. The $\lambda/2$ design is evident from the electric field nodes shown in Supplementary Fig. 5b, with the maximum field enhancement obtained at the active $WSe_2/MoSe_2$ heterobilayer.

Supplementary Fig. 6 shows the simulated reflectance cavity mode in our structure with different numbers of top DBR pairs. A higher number of top pairs is related to an increase in the Q factor. In contrast, the electric field enhancement obtained at the active area is not monotonically dependent on the number of pairs, with maximum enhancement at 5 pairs (Supplementary Fig. 6b). In order to meet a trade-off between field enhancement and transparency, we grew 6 pairs of top DBR mirrors on both devices A and B. Supplementary Fig. 7c displays the obtained quality factor for the full structure as represented in Supplementary Fig. 5, comprising all 2D material layers. For 6 top DBR pairs, a quality factor of approximately 200 was simulated. The reduced quality factor measured in our full cavity (Supplementary Fig. 4) with respect to the ideal simulated structure (Supplementary Fig. 7) is a result of the presence of non-uniformities in the heterostructure area, such as dielectric bubbles, neighboring bulk layered materials or metal contacts.

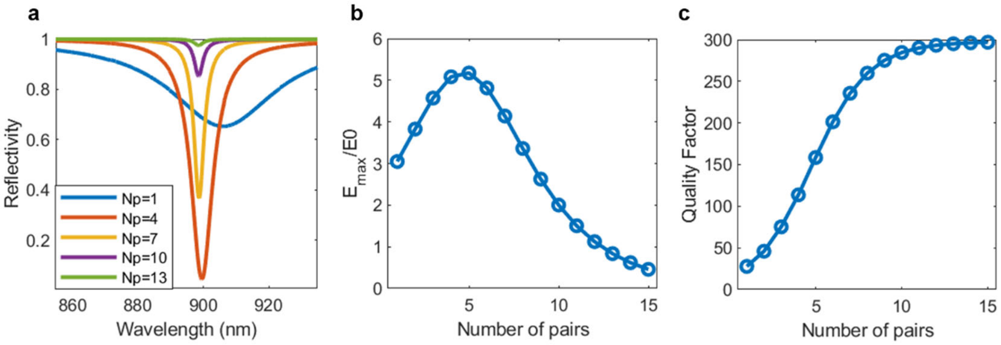

Supplementary Fig. 6. a, Simulated reflectance dip for a cavity structure centered at 900 nm. The Q factor increases monotonically with the number of top DBR pairs (N_P). **b**, Maximum electric field enhancement with respect to the number of top DBR pairs. The highest enhancement is obtained with approximately 5 top DBR pairs. **c,** Quality factor with respect to N_P.

The simulated quality factor is degraded with respect to ideal structures based on a SiO_2 cavity and DBR mirrors due to the presence of absorbing 2D layers. In particular, we have investigated the lowering of the Q factor with respect to the presence of top and bottom graphene gates. Supplementary Fig. 7 shows the Q factors with respect to the number of top DBR pairs without graphene layers (Supplementary Fig. 7a), as well as with one (Supplementary Fig. 7b) and two (Supplementary Fig. 7c) graphene gates.

The use of bottom and top graphene gates is crucial for the field-dependent modulation of the exciton-cavity tuning by the quantum-confined Stark effect. However, the presence of graphene in the stack results in a decrease of the Q factor by more than one order of magnitude, thus explaining the simulated values in Supplementary Fig. 6 as well as our experimental observations.

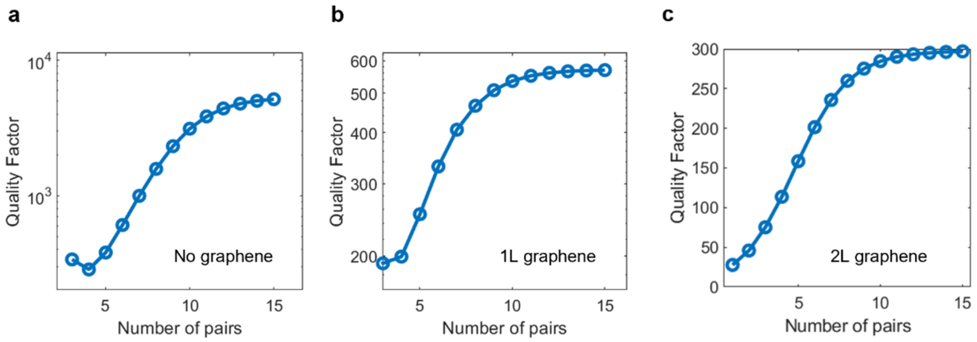

Supplementary Fig. 7. a-c, Quality factor of the structure in Supplementary Fig. 6 with respect to the number of top DBR pairs without graphene layers (a), with a single graphene gate (b), and with two graphene gates (c). The obtained Q factor decreases by more than an order of magnitude with the introduction of graphene.

5. Field-dependent lifetime in device A

We obtained the lifetime data in Fig. 2 in the main text by exciting our IXs in the full-cavity structure with a picosecond laser as described in the Results section. The complete field-dependent data is shown in Supplementary Fig. 8a. We used a relatively low average power of 50 nW at a repetition rate of 1 MHz, obtaining decay curves that are best characterized by single-exponentials (Supplementary Fig. 8b).

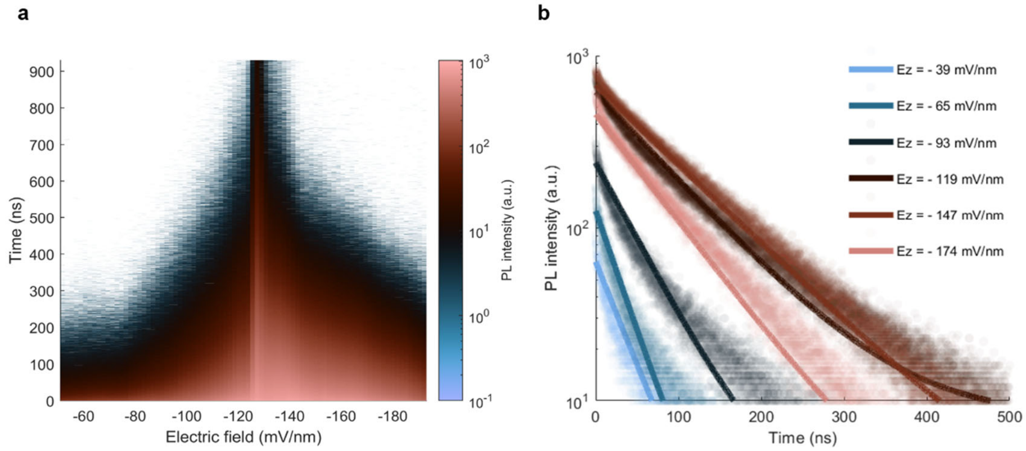

Supplementary Fig. 8. Field-tunable lifetime in device A. a, Field-dependent PL decay curves of IXs in the full-cavity structure, excited by a picosecond laser (1.93 eV) with repetition rate of 1 MHz and average power of 50 nW. **b,** Decay traces extracted from (a) at specific field values, highlighting the sizeable change with respect to the lifetime enhancement in the vicinity of -120 mV/nm. The lifetime data of Fig. 2 in the main text is obtained by fitting the data in (a) by single-exponential decays, as shown by the solid lines in (b).

6. Additional characterization of device A

All data reported in the main text was taken from the same position within the heterostructure area, which we refer to as point A. However, $MoSe_2/WSe_2$ heterobilayer samples are known to exhibit position-dependent variabilities of the interlayer exciton properties due to atomic

reconstruction[9]. To address this, we performed a spatial analysis of the IX emission across our heterostructure.

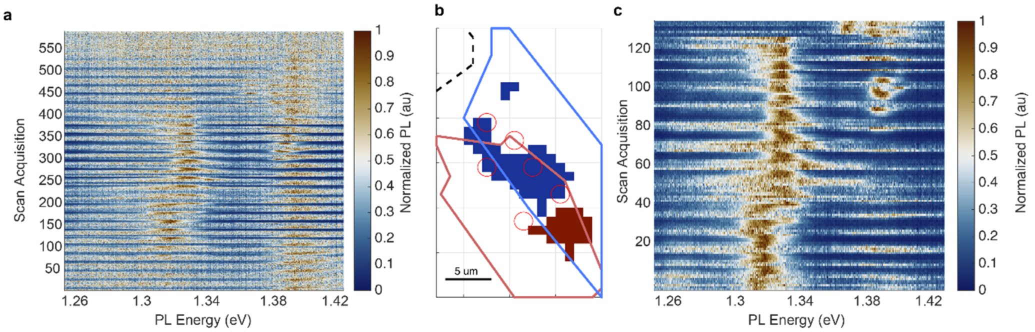

Supplementary Fig. 9. Spatial PL scan. a, Normalized spectra acquired while scanning the position of the objective around the heterostructure in an S-like scan path. Different positions are referenced to as "Spectrum #", as labelled on the y axis. **b**, Discretized spatial points showing integrated PL intensity larger than a threshold, and whose peak energy falls between the 1.28 eV and 1.315 eV (1.315 eV and 1.385 eV) for the red (blue) pixels. The red circles show the larger bubbles within and around the heterostructure. The blue (red) overlay show the WSe_2 ($MoSe_2$) flake, while the black dashed line shows the nearby electrical contact. **c**, Normalized spectra from the red and blue regions in panel b, shown in a sequence.

Supplementary Fig. 9a shows the spectroscopic luminescence acquired while scanning the objective around the heterostructure. In this plot, each point on the y axis (labeled "Spectrum #") represents a different pixel in the S-shaped scan across the heterostructure area in Fig. 3b. This type of plot allows us to recover the main energetic components of the IX peaks across a 2D area projected on a single axis[10]. Applying basic masking rules, such as minimum integrated PL, and peak energy falling within a certain range, we can accurately recover the heterobilayer shape and position, as confirmed by the overlays and the colored pixels shown in Supplementary Fig. 9b. In Supplementary Fig. 9c we show the spectra points acquired during the scanning which satisfied the masking criteria. This analysis displays a good homogeneity of the spectroscopic features observed within the whole heterostructure.

Throughout the scan, we observe two main peaks centered around 1.313 eV and 1.325 eV, with an energy difference of roughly 12 meV. The origin of these two peaks cannot be explained by spin-singlet and spin-triplet transitions, due to the smaller detected energy difference with respect to the singlet-triplet energy difference (~25 meV)[11,12]. Instead, this energy difference is most likely induced by the variability of the strain environment around the analysis spot, as confirmed from our spatial analysis. In fact, the 1.313 eV (1.325 eV) peak is dominant in the lower (upper) part of the heterostructure, indicated by the red (blue) pixels in Supplementary Fig. 9b. This plot also shows how the red (blue) cluster is free of (surrounded by) dielectric bubbles, indicated by red circular overlays. This is in agreement with previous experimental studies finding energy shifts of up to 20 meV in $WSe_2/MoSe_2$ under tunable tensile strength[13].

To address the presence of two IX peaks, we performed the same set of measurements as in the main text for another position within the heterostructure, which we refer to as point B. The Stark effect, the reflectance dip, the integrated PL and lifetime of the IXs in point B are reported in Supplementary Figs. 10a-d, respectively. The position of the main cavity dip is located at 1.38 eV, as for point A. Supplementary Figs. 10c-d show an enhancement of both PL intensity and lifetime when the exciton-cavity mode matching condition is achieved, confirming our

results in the main text. However, due to a different IX peak composition in point B with respect to point A (Fig. 1), the combined PL intensity and lifetime enhancement is obtained around 90 mV/nm in the former case.

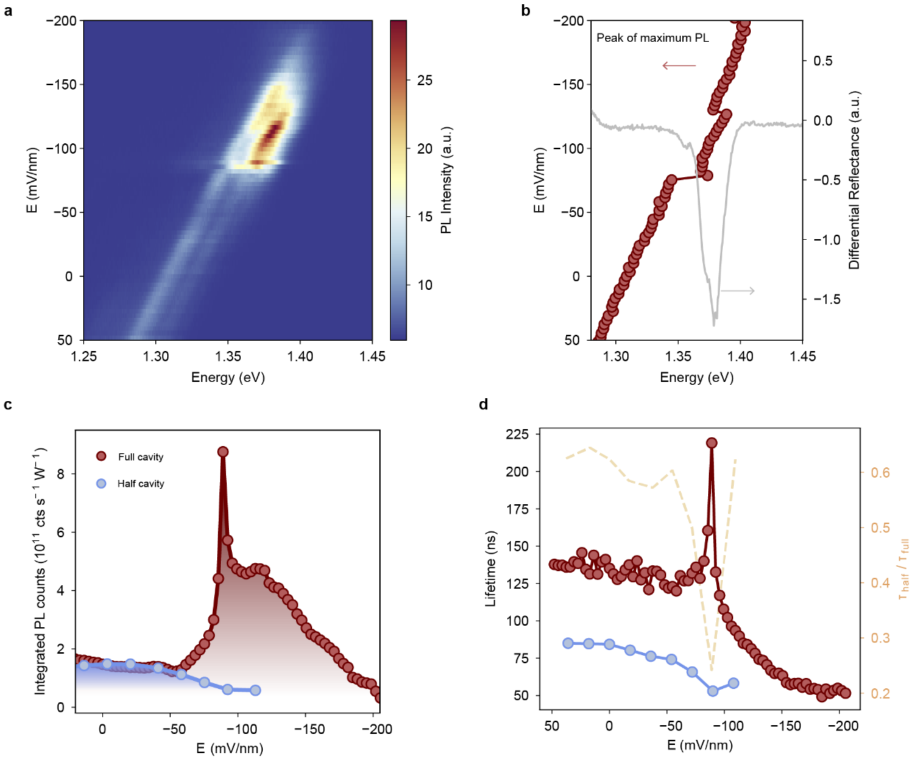

Supplementary Fig. 10. Field-tunable IXs in position B. a, Interlayer exciton PL spectra with respect to the applied vertical electric field E_z, obtained by exciting the structure with a laser power of $1\ \mu$W. Two main interlayer peaks, IX^1 and IX^2, are present at any given field, shifting linearly with E_z. A region of higher intensity is observed in the vicinity of the cavity mode energy (1.38 eV). **b,** Field-tunable position of the highest-emitting IX peak energy (red). A rigid shift of the brightest peak to IX^2 is found at the exciton-cavity tuning condition. The cavity mode is highlighted by superimposed differential reflectance dip (grey). **c,** Total integrated IX PL intensity with respect to E_z for the half-cavity (blue) and full-cavity (red) structures. In the former case, the integrated intensity decreases monotonically for positive electric fields. In the full-cavity case, a sharp rising peak at $E_z \simeq -90\ \mathrm{mV/nm}$ is followed by a more gradual decrease at higher field strengths. A 16-fold enhancement is observed from the half to the full cavity structure at resonance. **d,** Field-dependent lifetime in the half-cavity (blue) and full-cavity (red) structures. The lifetime enhancement at the exciton-cavity coupling condition is followed by a monotonically decreasing trend for higher fields, in agreement with the field-dependent lifetime of uncoupled IXs[14,15]. The inhibition of the spontaneous emission rate between half and full-cavity structures (τ_{half}/τ_{full}) is highlighted in yellow. The lifetime enhancement is present even in the off-coupling condition due to the inhibition of photonic modes for in-plane IX transition dipoles, as discussed in the main text as well as in Supplementary Note 10.

We refer to the two aforementioned peaks as IX^1 and IX^2. Far from resonance ($E_z > -50\ \mathrm{mV/nm}$), the lower-energy peak IX^1 appears as the brightest species. Instead, when in the vicinity of resonance, the higher-energy peak IX^2 dominates the spectrum (Supplementary Fig. 10a). At lower fields ($E_z < -130\ \mathrm{mV/nm}$), the strongest emission shifts again to IX^1. This is due to the fact that, while both peaks shift linearly with increasing E_z, IX^2 is the first one to reach the cavity transparency, for lower fields with respect to IX^1, due to its higher energy.

Supplementary Figs. S10c-d also display (in blue) the field-dependent integrated IX intensity and lifetime for the half-cavity case, in order to evidence the obtained Purcell inhibition and the absence of peaks in intensity or lifetime in this situation. This data is the

same as the one presented in Supplementary Fig. 2. Regarding the full-cavity structure, we note that no increase in PL intensity is recorded below resonance (Supplementary Fig. 10c). This is due to the fact that, while a change in the photonic density of states is still responsible for a sizeable Purcell inhibition below resonance, the transparency of the cavity structure is not affected – or only slightly reduced – by the top PECVD growth in this energy range. Thus, since no coupling is obtained for such fields, the emitted radiance is comparable for half-cavity and full-cavity structures.

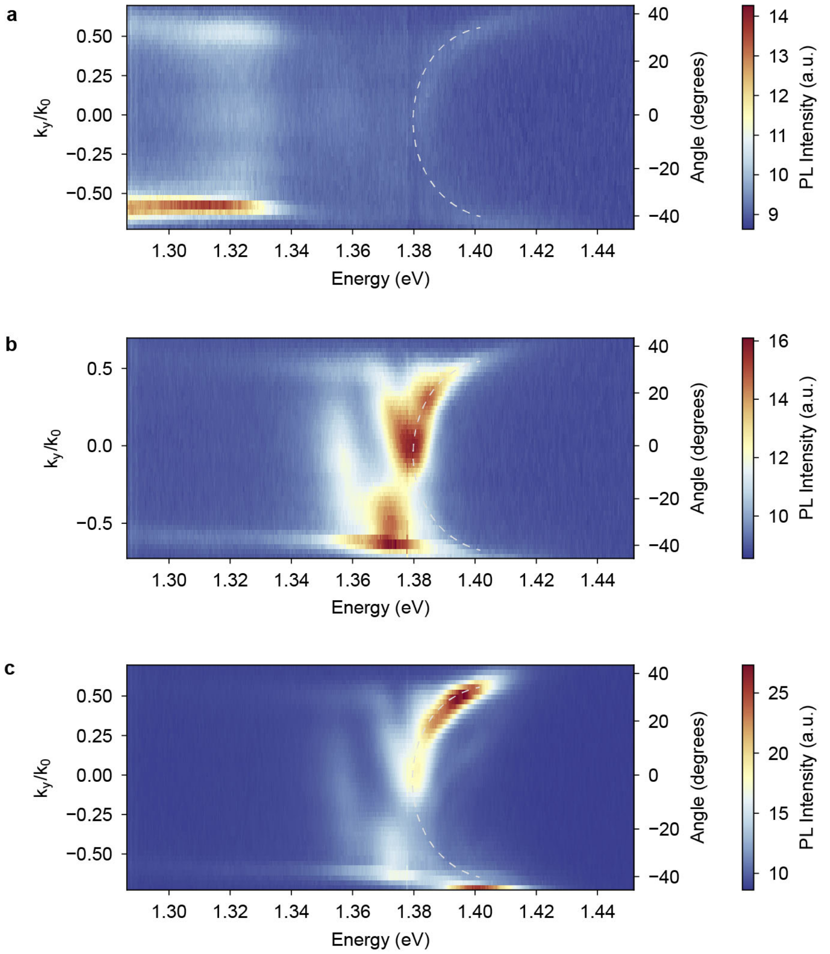

Supplementary Fig. 11. Momentum-resolved emission of IXs in point B of device A at high power. a-c, Angular emission of IXs in point B of device A at electric fields $E_z \simeq 100\ \mathrm{mV/nm}$ (a), $-90\ \mathrm{mV/nm}$ (b), and $-150\ \mathrm{mV/nm}$ (c), respectively. All measurements were obtained exciting the structure with $0.9\ \mathrm{mW}$ of laser power. Before resonance, high-angle emissions dominate the spectrum, together with a non-negligible signal around zero momentum. When moving from low-angle (b) to high-angle resonance (c), a progressive quasi-parabolic shift of the PL emission is observed, as for point A (Fig. 4 in the main text).

Furthermore, Supplementary Fig. 11 shows the momentum-resolved IX emission in energy from point B at three different electric fields, namely before resonance (Supplementary Fig. 11a), at low-angle resonance (Supplementary Fig. 11b), and at high-angle resonance (Supplementary Fig. 11c). Here, we used a higher optical excitation power (0.9 mW), with respect to point A in the main text (1 μW). Nonetheless, the increase in low-angle emission in Supplementary Fig. 11b at low-angle resonance is aligned with that of Fig. 4b of the main text. The coexistence of both low and high-momentum components before resonance is aligned with

the result of transfer-matrix and FDTD simulations, as further detailed in Supplementary Note 8. The quasi-parabolic trend at cavity resonance is evidenced in Supplementary Fig. 11c by a super-imposed dashed line. These results indicate that the observed tunable IX dispersion is obtained at different positions within the heterostructure and independently on the excitation power.

7. Characterization of device B

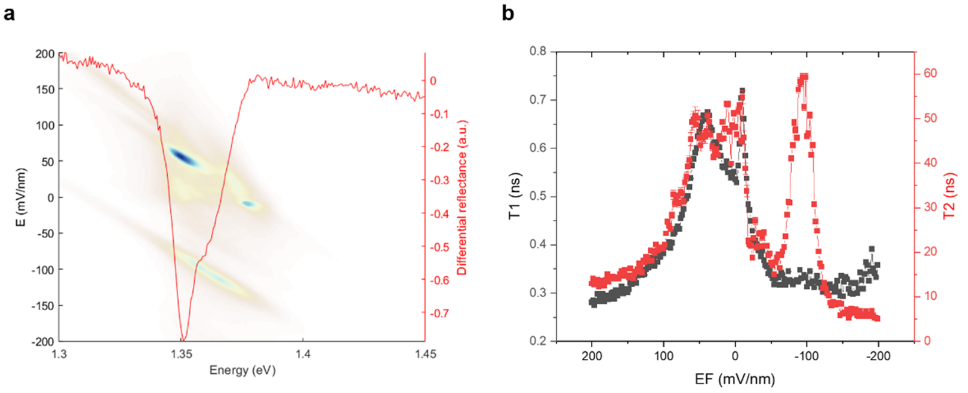

Supplementary Fig. 12. a, Field-dependent IX PL intensity from device B in the full-cavity structure. The Stark effect reveals multiple peaks with the same linear field dependence (static dipole moment). The differential reflectance signal in red, taken at the same position within the structure, exhibits a main dip around 1.36 eV. We observe a sizeable increase of PL intensity for all peaks when entering in the range of the cavity mode. **b,** Field-dependent IX lifetime in device B. A bi-exponential fit is used to extract short (T1) and long (T2) decay times. T2 exhibits a five-fold increase in correspondence of the exciton-cavity resonance for the IX_{c1} and IX_{c2} clusters of peaks, corresponding to electric fields centered at -50 mV/nm and 100 mV/nm, respectively. A sizeable increase in T1 is also present for the IX_{c1} peaks.

We performed the full characterization of the device B (Supplementary Fig. 1), as done in the main text for device A with R-type stacking, in order to replicate our results in a second heterostructure with H-type stacking. The measurements of the twist angles and stacking types are further detailed in Supplementary Note 11.

Supplementary Fig. 12a displays the field-dependent PL intensity emitted from device B when excited with a continuous-wave (pulsed) laser at a power of approximately 1 μW (70 nW at 1 MHz) and a photon energy of 1.93eV for Stark effect (TRPL) measurements. The differential reflectance at the same position (Supplementary Fig. 12a, red line) reveals a cavity mode centered around 1.36 eV. We observe an increase of IX PL when the condition of exciton-cavity mode matching is achieved. The different IX peaks in the continuous-wave PL measurements can be grouped into two main clusters based on the electric field value where their PL emission enhancement is obtained. Therefore, we identify two main clusters of peaks, IX_{c1} and IX_{c2}, as indicated in Supplementary Fig. 12a. The two clusters correspond to a PL enhancement obtained for the field ranges $100\ \mathrm{mV/nm} > E_z > 0\ \mathrm{mV/nm}$ (IX_{c1}) and $-50\ \mathrm{mV/nm} > E_z > -110\ \mathrm{mV/nm}$ (IX_{c2}). The presence of multiple IX peaks within a wide range of energies is known to be commonly present in $MoSe_2$/WSe_2 heterobilayers based on the moiré superlattice[16–18] as well as the presence of disorder and defective states within the structure[19,20]. The specific role and origin of such a variety of peaks in these structures is well documented in the literature and is outside the scope of this work.

Supplementary Fig. 11b shows the field-dependent lifetimes of IXs in device B. While for device A the measured decays are best fit by single-exponential curves, for device B all traces

are best fit by bi-exponential decays, giving short (T1) and long (T2) decays. We note that we are collecting all lifetimes from all IX peaks involved at any given field. Nonetheless, we can consider that, in the conditions of exciton-cavity mode matching, the coupled IXs contribute most to the measured signal. With this assumption, we note that two main electric field regions exhibit an increase of the long decay channel, corresponding to the regions of PL enhancement of peak clusters IX_{c1} and IX_{c2}. A five-fold increase in the measured lifetime is obtained at the points of exciton-cavity matching. This is qualitatively aligned with the lifetime increase observed for device A in Fig. 2 in the main text.

We note that the IX_{c1} cluster exhibits an enhancement of PL intensity and long decay T2, while no significant increase in T1 is reported (Supplementary Fig. 12b). Thus, fast non-radiative decay dynamics of IX_{c1} are not affected by IX-cavity tuning at low excitation power, indicating that the lifetime trend enhancement is not related to the decrease of non-radiative decay rates. This suggests that the increase of the single-exponential decay channel in device A is also not related to a modulation of the non-radiative decay rates. On the other hand, the long radiative decay, which is strongly modulated by the exciton-cavity detuning via electrical means.

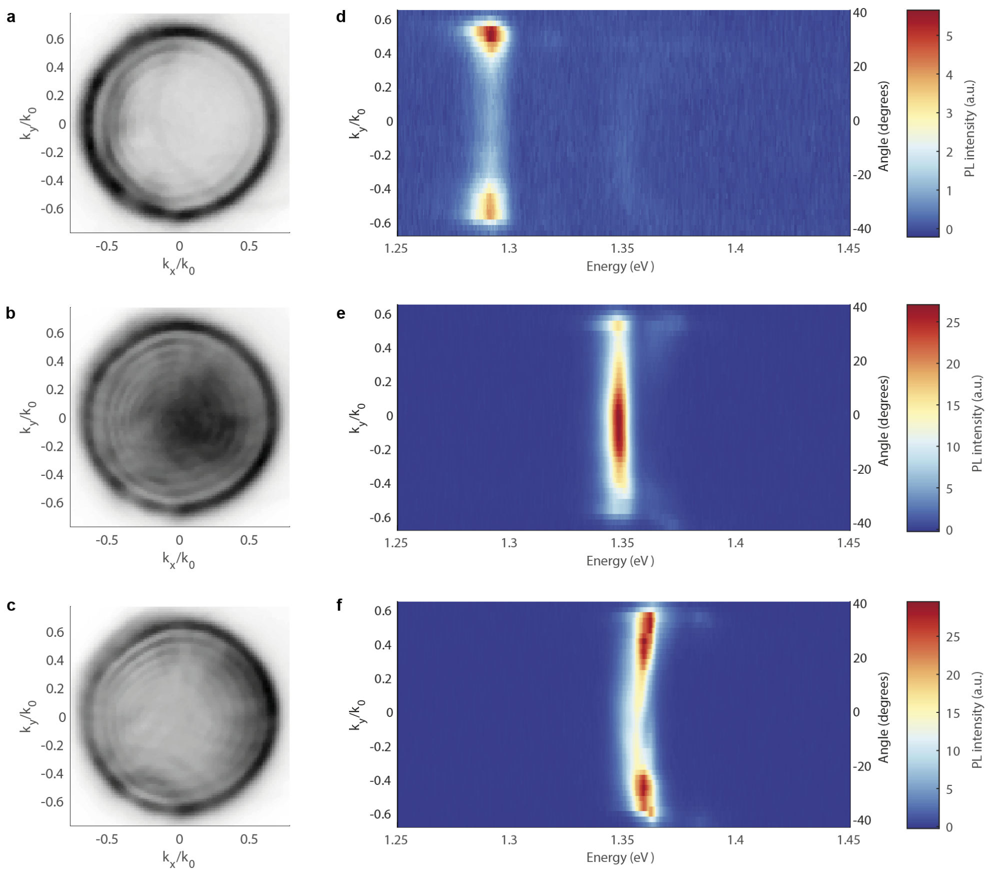

Supplementary Fig. 13. a-c, Back-focal-plane (BFP) images of the emitted IX PL intensity from device B at electric fields of 200 mV/nm (a), -55 mV/nm (b), and -80 mV/nm (c). As for device A (Fig. 3 in the main text), we are showing BFP images before resonance (a), at low-angle resonance (b) and at high-angle resonance (c), respectively. In this case, we are considering the resonant condition of the highest-intensity PL peaks, indicated in Supplementary Fig. 10 as IX_{c1}. **d-f,** Angular emission in energy of IXs in device B at fields of 200 mV/nm (d), -55 mV/nm (e), and -80 mV/nm (f). The dispersion in energy before and after resonance is highly compatible with that obtained from device A (Fig. 4 and Supplementary Fig. 11). A strong dominant low-angle component is visible at the first resonant condition (e), aligned with

all other measurements as well as with transfer-matrix simulations (Supplementary Note 8).

We further investigated the momentum-resolved IX emission with respect to the applied electric field. Supplementary Figs. 13a-c display the raw back-focal-plane (BFP) images of the IX emission from device B at three different electric fields of interest. The regions of reference are identified in the same way as for device A in the main text. The chosen fields correspond to the conditions of before-resonance (200 mV/nm), low-angle resonance (-55 mV/nm), and high-angle resonance (-80 mV/nm) for IX_{c1}. A dominant high-angle emission is obtained in the before-resonance condition, with non-negligible low-angle components. In resonance, low-angle components are enhanced (Supplementary Fig. 13b), with dominant emissions transitioning back to higher angles with increasing field magnitudes (Supplementary Fig. 13c). Supplementary Figs. 11d-f show the angular emission obtained from the k_y component of the IX PL emission with respect to energy. The characteristic shapes of the cavity coupled IX dispersion are displayed in Figs. 4a-c in the main text and are reproduced here for device B, with a quasi-parabolic trend at resonance.

We can also appreciate a faint, but non-negligible, quasi-parabolic signal above 1.35 eV when in the before-resonance condition (Supplementary Fig. 13d). This is consistent with our simulations of far-field emission scaled by a Lorentzian factor, as shown in Supplementary Note 9. In particular, Supplementary Fig. 16a displays the emission of an ideal in-plane dipole with a full-width half-maximum (FWHM) of 10 nm centered far from resonance (1.30 eV), which still allows coupling at resonant energies due to its non-vanishing tail.

With the characterization of device B, we confirmed our observations on the electrically-tunable cavity-coupling of IXs with monolithic systems in a second heterostructure with H-type stacking (Supplementary Note 11). The presence of a different distribution of IX peaks in device B, compared with the signatures of device A, suggests that the observed field-dependent IX emission and dynamics are obtained despite device-dependent variabilities. Thus, we can conclude that the obtained manipulation of the IX emission and dynamics in our work is based on the cavity coupling of in-plane IX transition dipoles, with a behavior that is qualitatively independent of sample-related and position-related variabilities.

8. Simulations of cavity-coupled dipolar emission

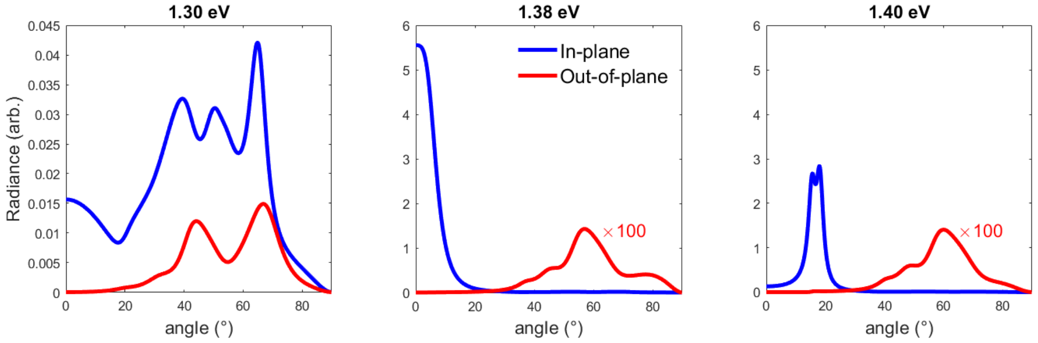

Supplementary Fig. 14. Calculated radiance of an incoherent in-plane (blue) and out-of-plane (red) classical dipole in the cavity system for three representative energies.

The calculation of the radiance (i.e., flux per solid angle) provides a measure of the angular distribution of the power emitted by a dipole into the far field. This depends on the orientation

of the dipole, the photonic environment, and emission wavelength. Using a transfer-matrix-based method suitable for unpatterned multilayer stacks (Ansys Lumerical STACK), where the exciton emission is modelled as classical dipole radiation, the radiance emission profile is calculated at the three representative energies for an incoherent in-plane (blue line) and out-of-plane (red line) oriented dipole (Supplementary Fig. 14). The three energies are chosen with respect to a cavity mode located at 1.38 eV, as in Fig. 4 in the main text, representing the conditions of before resonance (1.30 eV), in-plane dipole low-angle resonance (1.38 eV), in-plane dipole high-angle resonance (1.40 eV). Here, the angle is measured from the surface normal (taken to be the z direction). The in-plane dipole emission is given as the incoherent sum of dipoles oriented in the x and y directions.

At all three energies, the in-plane dipole emission is significantly greater than the out-of-plane emission at low angles. In contrast, the out-of-plane dipole preferentially emits into larger angles above ~20 degrees. At energies close to the microcavity resonance (1.38 eV and 1.40 eV), the emission of an in-plane dipole is about two orders of magnitude greater than an out-of-plane dipole due to efficient coupling into the Fabry-Perot mode, and peaks strongly at lower angles. As the energy is increased, the Fabry-Perot mode moves to higher momenta (see Fig. 4d in the main text), consequently causing the radiance to peak at higher angles. Below resonance (1.30 eV), the in-plane dipole emits over a greater spread of angles, and the emitted power is comparable between the two dipole orientations.

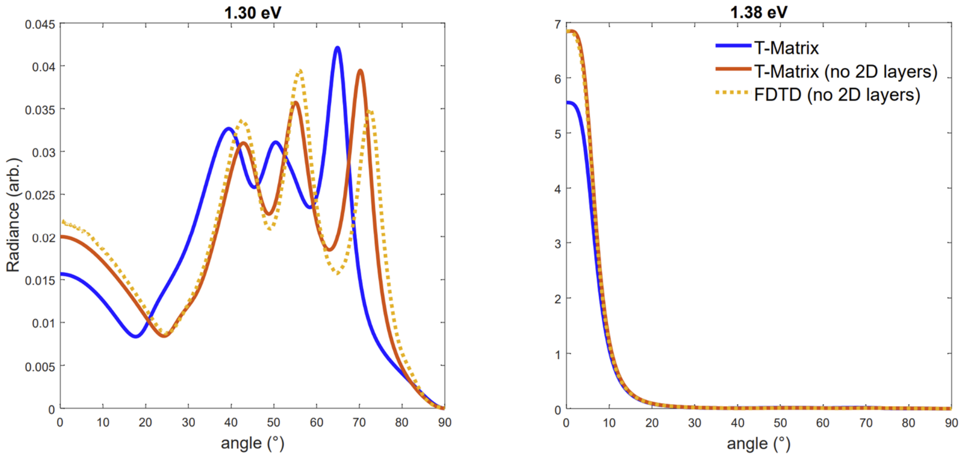

Supplementary Fig. 15. Comparison of different models and computational methods for the calculated radiance of an incoherent in-plane classical dipole in the cavity system for off- (a, 1.30 eV) and on-resonance (b, 1.38 eV). For both plots, we considered a transfer matrix method comprising the full heterostructure (blue line), as well as transfer matrix (red line) and FTDT simulations (dashed yellow line) for a stack with only the hBN components, without monolayers.

To confirm our theoretical results, we have also utilized the finite-difference time-domain (FDTD) method[21] (using MEEP[22]). Cylindrical coordinates are used to exploit the in-plane symmetry of the cavity system, reducing the problem to a 2D simulation. The Poynting vector in the far field is calculated using MEEP's near-to-far field transformation, from which the radiance can be calculated. Due to the challenge of meshing 2D materials, we employ a simplified model where the monolayers, namely the graphene gates and the TMDCs, are not taken into account in the electromagnetic calculation. We note that the bulk bottom and top hBN layers are always considered. In Supplementary Fig. 15, we compare the radiance of an incoherent horizontally-aligned dipole calculated with the simplified model using the transfer-matrix (T-matrix) method (orange line) and FDTD (dotted yellow line). We also show results

for the full model, including 2D layers, calculated using the T-matrix method (blue line, same model as the one used in Supplementary Fig. 14). Excellent agreement is found between the two computational methods, and there is qualitative agreement between the two different models.

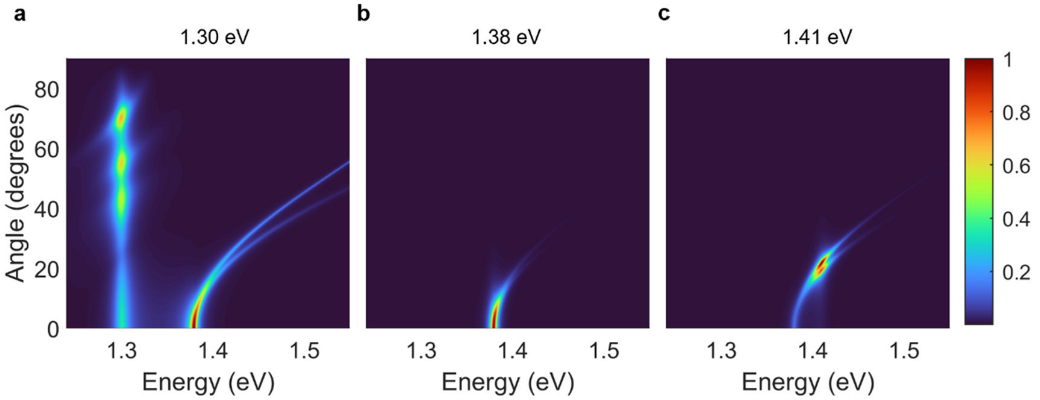

Supplementary Fig. 16. Normalized calculated radiance integrand of an incoherent in-plane dipole in the cavity system (as in Fig. 4d) scaled by a Lorentzian centred at 1.30 eV (a), 1.38 eV (b) and 1.41 eV (c). The energies of reference are chosen based on the experimental results displayed in Fig. 4 in the main text for device A.

All simulations previously shown in this section, as well as those presented in Fig. 4 in the main text, provide the angle-dependent emission patterns of in-plane and out-of-plane classical dipoles, aiming at understanding the role of IX transition dipoles in the measured weak coupling regime. As discussed in the main text, only in-plane transition dipoles couple effectively with our cavity structure, thus explaining the significant change in emission pattern with respect to the applied electric field. In order to mimic the field-dependent data from simulations, we convolute the transfer-matrix data of in-plane dipole emission with a Lorentzian function centered at a given energy with a FWHM of 10 nm to mimic an ideal single IX PL lineshape emission. By shifting the center of the Lorentzian function in energy, we obtain an idealized single in-plane excitonic transition dipole with tunable emission energy. In particular, Supplementary Fig. 16 shows the in-plane radiance scaled by a Lorentzian function centered at 1.30 eV (Supplementary Fig. 16a), 1.38 eV (Supplementary Fig. 16b) and 1.40 eV (Supplementary Fig. 16c), corresponding to the above mentioned energies of interest for our system.

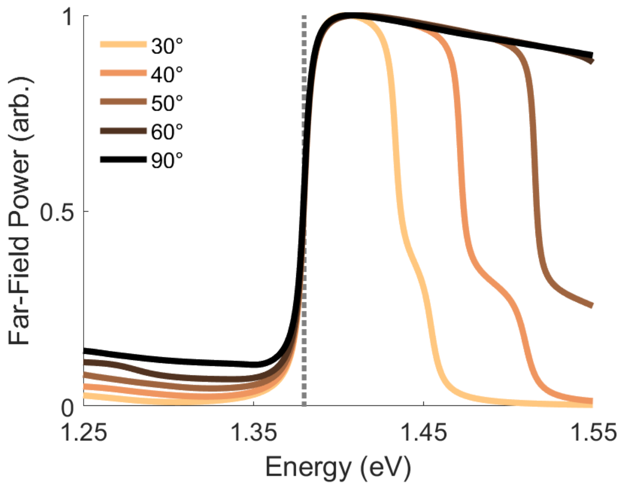

Supplementary Fig. 17. Calculated far-field power spectrum of an in-plane classical dipole within the cavity system for varying integrated angular ranges, mimicking the impact of the numerical aperture of a detector. At 90° (black line) all far-field power is collected.

In realistic experiments, a substantial percentage of the far-field emission is not collected due to the finite aperture of the microscope objective. To mimic this effect, we integrate the far-field power density (calculated using the "stackdipole" command from Ansys Lumerical STACK) over a limited range of angles to obtain the total power emitted by an incoherent horizontally-aligned dipole in our cavity system (Supplementary Fig. 17). In particular, integrating up to an angle of 40° corresponds to the numerical aperture of 0.65 used in our experiment. We find an asymmetric spectrum centred on the microcavity resonance (vertical grey-dotted line) in the limit that all emitted light is collected (black line). The increased power emission above resonance is a consequence of the Purcell effect via the coupling of energy into the microcavity mode. In fact, the power spectrum here has a characteristic asymmetric profile that is well-known for the spontaneous emission enhancement of an in-plane dipole in a dielectric microcavity[23] (Supplementary Fig. 18). Since the local density of states is related to the power irradiated by our dipoles, we explain the observed asymmetry by the change in photonic mode density for in-plane dipoles at resonant conditions, as further discussed in Supplementary Note 9. Below resonance, there is a straightforward increase in total measured power as the collection angle is increased. This is a consequence of the broad range of angles that the systems emits into at lower energies (see Supplementary Fig. 14a). For decreasing numerical apertures, the high-energy tail of the power spectrum decreases in magnitude and a more symmetric spectrum is recovered. This is because, at energies well above resonance, a large numerical aperture is needed to collect the directed emission into high angles due to the microcavity dispersion (see Supplementary Fig. 14c, as well as Fig. 4d in the main text). If a given numerical aperture cannot capture this sharp emission, then only the lower power densities are collected and there is consequentially a rapid drop in the integrated power as a function of the emission energy.

9. Transition dipole orientation and weakly-coupled IX lifetime

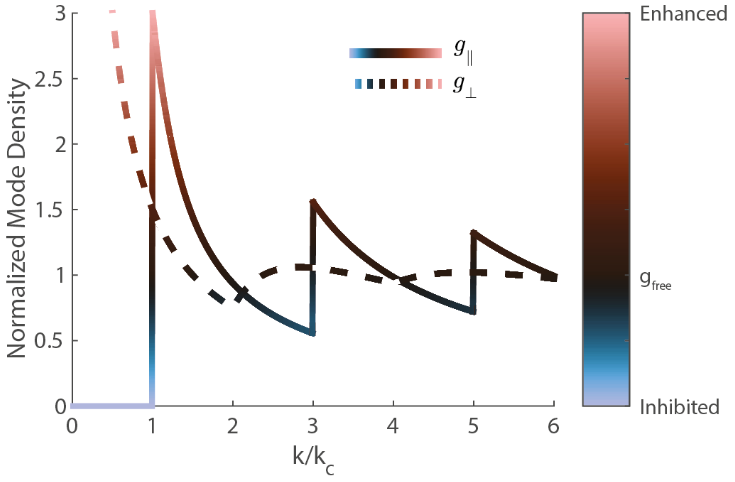

Supplementary Fig. 18. Mode density for ideal emitting dipoles oriented parallel ($g_{//}$) and perpendicular ($g_{\perp}$) to the plane of an idealized planar cavity, normalized to the free dipole g_{free}[24].

Given the coexistence of in-plane and out-of-plane IX transition dipoles in our structures, we must consider the optical mode density change with respect to the emitted photon energy for both situations. Here, we employ a simplified model with idealized in-plane and out-of-plane emitting dipoles to interpret the recorded IX dynamics (Fig. 2c in the main text).

Supplementary Fig. 18 shows the normalized mode density for an idealized planar cavity with dipoles oriented parallel ($g_{//}$) and perpendicular ($g_{\perp}$) to the plane, considering mirrors perfectly reflecting over all angles. Although we follow the same methodology as in Ref. [[25]], we adopt here, for convenience, a nomenclature based on the plane of the layers rather than the perpendicular axis. A strong inhibition of modes for in-plane dipoles $g_{//}$ with $k < k_C$ is followed by a sharp increase in mode density for $k_C \leq k \leq 2k_C$. On the other hand, the mode density of out-of-plane dipoles $g_{\perp}$ undergoes smooth variations in the vicinity of k_C. Following Fermi's golden rule, the emission rate of an emitting dipole is directly proportional to its mode density. Thus, the strong inhibition of modes with respect to the free dipole g_{free} for in-plane transition dipoles $g_{//}$ in $k < k_C$ is directly related to an increase in the corresponding exciton lifetime, while for $k \geq k_C$ a sharp decrease in their lifetime is expected. This is qualitatively aligned with the measured IX lifetime with respect to the electric field reported in Fig. 2c in the main text. Therefore, the peak in lifetime for $E < E_C$ for our IXs is related to the weak coupling of in-plane dipoles, which induces a strong discontinuity of their mode density around E_C.

When considering a lossy cavity, a smoother variation in mode density is obtained, as shown in Ref. [[24]]. Moreover, the above considerations do not take into account the linewidth of the measured IX emission. However, such a qualitative approach is sufficient to understand the mechanism behind the significant variation of IX lifetime we record in the vicinity of the exciton-cavity mode matching condition.

10. Experimental setup

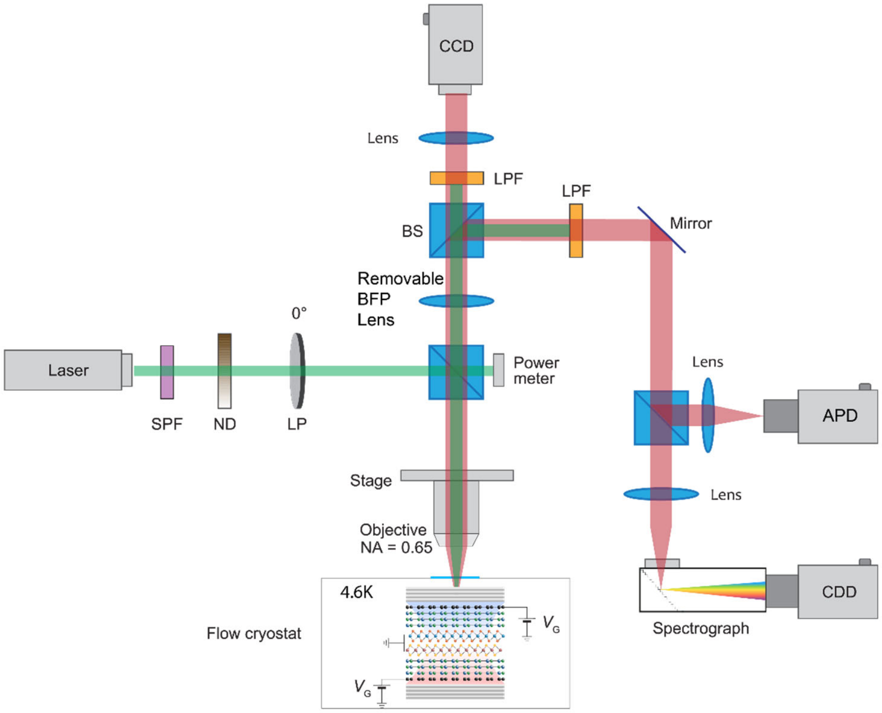

Supplementary Fig. 19. Schematic of the experimental setup used in this work.

Sample characterization was conducted in a He-flow cryostat in vacuum at a temperature of 4.6 K. Optical measurements were performed in a custom-built confocal microscope shown in Supplementary Fig. 19. Interlayer excitons were photogenerated with a diode laser (Picoquant, LDH-IB-640-M), which can be electronically driven both in CW and pulsed modes. Laser spectral emission is cleaned of spourious signals using a short-pass filter (Thorlabs FESH650), and variably attenuated by an ND filter wheel, and finally linearly polarized with a suitable polarizer. Both excitation and detection are focused on the sample by an NIR objective (Olympus LCPLC50XIR), focusing the excitation spot with a full-width half maximum of roughly 1.2 µm. Detection and excitation paths are split with a 50:50 beamsplitter. Spectroscopy measurements are conducted focusing the PL image, filtered from laser excitation with a longpass filter (Thorlabs FELH800), on the spectrometer slit. The spectrometer (Princeton Instruments SpectraPro 500) diffracts light according to the photon energy, and shines it on a CCD camera (Princeton Instruments, Blaze 400-HR/HRX) for recording. Spatial resolution across the sample is achieved by spatially scanning the objective with a piezo-scanning stage (Mad City Labs Nano-T). Differential reflectance is measured by coupling the excitation path to a wavelength-stabilized white light source (Thorlabs SLS202L), and using the same setup for spectroscopy measurements. Time-resolved photoluminescence spectroscopy is conducted by directing part of the PL via a beamsplitter to a single-photon detection module (SPDM) (Excelitas Technologies, SPCM-AQRH-16). The SPDM output is connected to a time-correlated single-photon counting module (PicoQuant, PicoHarp 300), which logs the arrival time of each photon with a 12ps r.m.s. resolution. The temporal resolution of our system is limited by the SPDM, which features a single-photon timing resolution of roughly 350 ps. IX cloud imaging is conducted directing part of the emitted PL

to a CCD camera (Andor Ixon Ultra). Back focal plane imaging and spectroscopy were performed by inserting an additional plano-convex lens between the objective and the focusing lenses of the camera systems.

SHG measurements were performed using an 80 MHz 180-fs Ti:Sa laser system (Coherent, Chameleon Ti:Sapphire) set at 900 nm emission wavelength. The fs laser polarization has been set with a Glan-Thompson crystal polarizer (Thorlabs, GTH5M-B) and chromatically filtered with a long-pass 700 nm edge filter. Linear Polarization for crystalline axis orientation was rotated with a lambda-half super-achromatic waveplate (SAHWP05M-700) mounted on a motorized rotation stage (Thorlabs, PRM1/MZ8). SHG spatial maps were performed splitting reflected excitation and SHG signal with a 650 nm edge dichroic mirror. The reflected signal was recorded with a standard photodiode for spatial reflectance, while the SHG signal was analyzed with a linear polarizer, parallel to excitation polarization, and further filtered with a 500 nm edge short-pass filter before being recorded by our spectrometer system.

11. Twist-angle estimation

We performed second harmonic generation (SHG) measurements to estimate the twist angle between the constituent layers[26]. As shown in Supplementary Fig. 20, we estimate twist angles of 1.7°±0.3° and 1.3°±0.4° for devices A and B, respectively. Furthermore, through SHG spatial mapping we retrieve the stacking order, which reveals R-type (H-type) stacking for device A (B) based on the constructive (destructive) signal interference at the heterobilayer overlapping region.

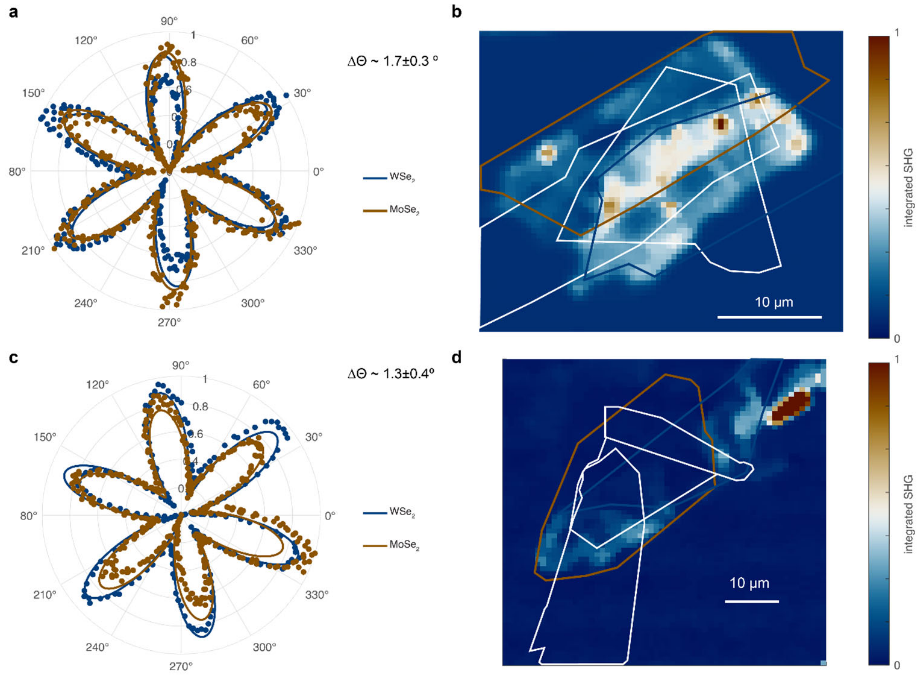

Supplementary Fig. 20. SHG measurements of device A and B. a,c,Polarization-dependent SHG measurement of the TMDCs monolayers (dots) with respective sinusoidal fits (solid lines) of devices A and B in panel (a) and (c), respectively. From sinusoidal fits, we estimate twist angles between the constituent monolayers of 1.7°±0.3° and 1.3°±0.4° for device A and B, respectively. **b,d,** SHG spatial mapping of device A and B in panel (b) and (d), respectively. For device A (B) the SHG intensity at the overlapping region is higher (lower) than that from the monolayer TMDCs, confirming R-type (H-type) stacking order.

REFERENCES

1. Ciarrocchi, A. *et al.* Polarization switching and electrical control of interlayer excitons in two-dimensional van der Waals heterostructures. *Nat. Photonics* **13**, 131–136 (2019).
2. Jauregui, L. A. *et al.* Electrical control of interlayer exciton dynamics in atomically thin heterostructures. *Science* **366**, 870–875 (2019).
3. Unuchek*, D. *et al.* Valley-polarized exciton currents in a van der Waals heterostructure. *Nat. Nanotechnol.* 1–6 (2019).
4. Gonzalez Marin, J. F. *et al.* Room-temperature electrical control of polarization and emission angle in a cavity-integrated 2D pulsed LED. *Nat. Commun.* **13**, 4884 (2022).
5. Heavens, O. S. *Optical Properties of Thin Solid Films*. (Courier Corporation, 1991).
6. El-Sayed, M. A. *et al.* Optical Constants of Chemical Vapor Deposited Graphene for Photonic Applications. *Nanomaterials* **11**, 1230 (2021).
7. Jung, G.-H., Yoo, S. & Park, Q.-H. Measuring the optical permittivity of two-dimensional materials without a priori knowledge of electronic transitions. *Nanophotonics* **8**, 263–270 (2019).
8. Hsu, C. *et al.* Thickness-Dependent Refractive Index of 1L, 2L, and 3L MoS_2, $MoSe_2$, WS_2, and WSe_2. *Adv. Opt. Mater.* **7**, 1900239 (2019).
9. Zhao, S. *et al.* Excitons in mesoscopically reconstructed moiré heterostructures. *Nat. Nanotechnol.* **18**, 572–579 (2023).
10. Blundo, E. *et al.* Localisation-to-delocalisation transition of moiré excitons in $WSe_2/MoSe_2$ heterostructures. *Nat. Commun.* **15**, 1057 (2024).
11. Joe, A. Y. *et al.* Electrically controlled emission from singlet and triplet exciton species in atomically thin light-emitting diodes. *Phys. Rev. B* **103**, L161411 (2021).
12. Yu, H., Liu, G.-B. & Yao, W. Brightened spin-triplet interlayer excitons and optical selection rules in van der Waals heterobilayers. *2D Mater.* **5**, 035021 (2018).
13. Ge, A. *et al.* Unraveling the strain tuning mechanism of interlayer excitons in $WSe_2/MoSe_2$ heterostructure. *Nanotechnology* **35**, 175207 (2024).
14. Tagarelli, F. *et al.* Electrical control of hybrid exciton transport in a van der Waals heterostructure. *Nat. Photonics* 1–7 (2023) doi:10.1038/s41566-023-01198-w.
15. Jauregui, L. A. *et al.* Electrical control of interlayer exciton dynamics in atomically thin heterostructures. *Science* **366**, 870–875 (2019).
16. Fang, H. *et al.* Localization and interaction of interlayer excitons in $MoSe_2/WSe_2$ heterobilayers. *Nat. Commun.* **14**, 6910 (2023).
17. Förg, M. *et al.* Moiré excitons in MoSe2-WSe2 heterobilayers and heterotrilayers. *Nat. Commun.* **12**, 1656 (2021).
18. Wang, X. *et al.* Moiré trions in $MoSe_2/WSe_2$ heterobilayers. *Nat. Nanotechnol.* **16**, 1208–1213 (2021).
19. Cai, H. *et al.* Interlayer donor-acceptor pair excitons in $MoSe_2/WSe_2$ moiré heterobilayer. *Nat. Commun.* **14**, 5766 (2023).
20. Mahdikhanysarvejahany, F. *et al.* Localized interlayer excitons in $MoSe_2$–WSe_2 heterostructures without a moiré potential. *Nat. Commun.* **13**, 5354 (2022).
21. Taflove, A., Hagness, S. C. & Piket-May, M. Computational Electromagnetics: The Finite-Difference Time-Domain Method. in *The Electrical Engineering Handbook* 629–670 (Elsevier Inc, 2005). doi:10.1016/B978-012170960-0/50046-3.
22. Oskooi, A. F. *et al.* Meep: A flexible free-software package for electromagnetic simulations by the FDTD method. *Comput. Phys. Commun.* **181**, 687–702 (2010).

23. Abram, I., Robert, I. & Kuszelewicz, R. Spontaneous emission control in semiconductor microcavities with metallic or Bragg mirrors. *IEEE J. Quantum Electron.* **34**, 71–76 (1998).

24. Yokoyama, H. & Ujihara, K. *Spontaneous Emission and Laser Oscillation in Microcavities*. (CRC Press, 1995).

25. Brorson, S. D. Eiectromagnetic Field Mode Density Calculated via Mode Counting. in *Spontaneous Emission and Laser Oscillation in Microcavities* (CRC Press, 1995).

26. Hsu, W.-T. *et al.* Second Harmonic Generation from Artificially Stacked Transition Metal Dichalcogenide Twisted Bilayers. *ACS Nano* **8**, 2951–2958 (2014).
