# Supplementary figures and images for: Enhancing interlayer exciton dynamics by coupling with monolithic cavities via the field-induced Stark effect
Source: Nat Nanotechnol. 2025 Jul 17;20(10):1412–8. doi: 10.1038/s41565-025-01969-2 (PMC12534180; doi:10.1038/s41565-025-01969-2)

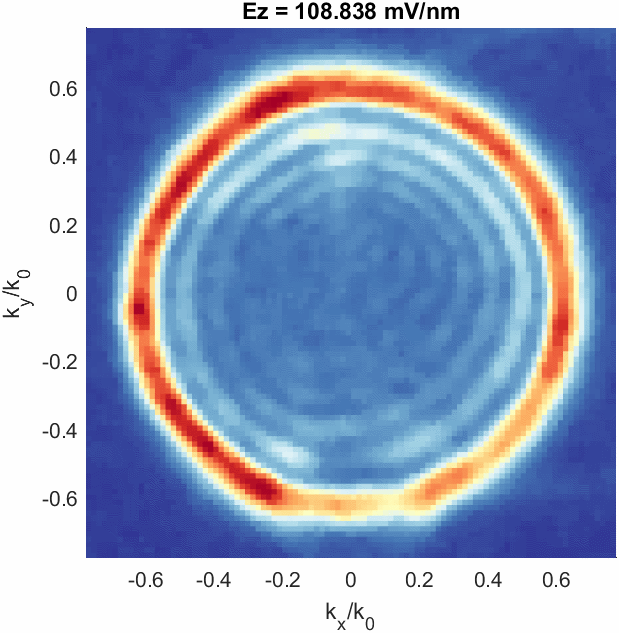

Supplement: Supplementary file 2 — Field-dependent BFP measurements of IX emission in device A. [file 41565_2025_1969_MOESM2_ESM.gif]

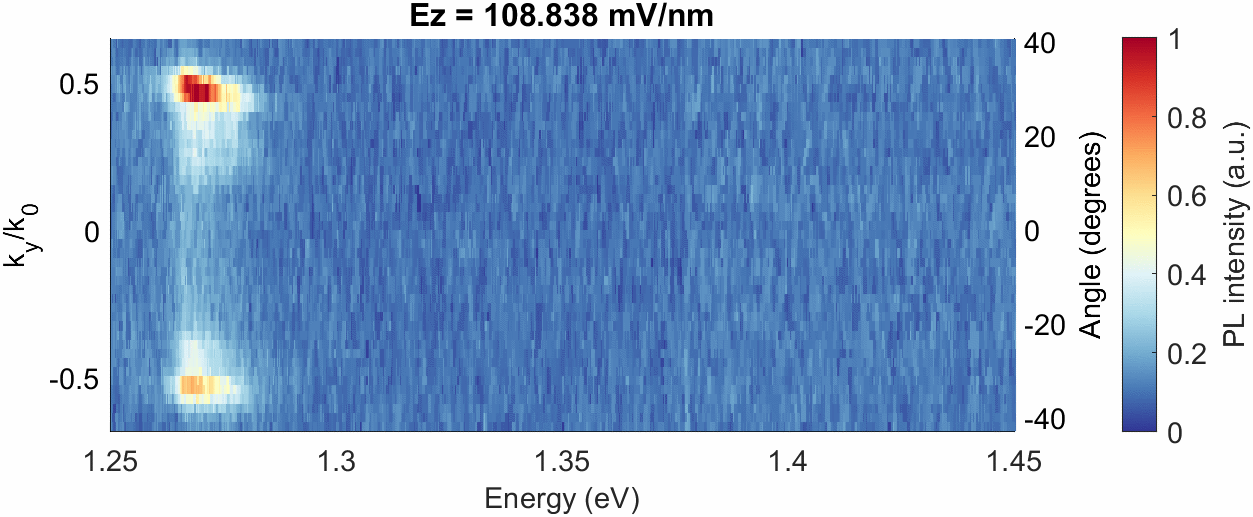

Supplement: Supplementary file 3 — Field-dependent energy-resolved measurements of IX angular emission in device A. [file 41565_2025_1969_MOESM3_ESM.gif]
